# Allelic variations in the *chpG* effector gene within *Clavibacter michiganensis* populations determine pathogen host range

**DOI:** 10.1371/journal.ppat.1012380

**Published:** 2024-07-19

**Authors:** Raj Kumar Verma, Veronica Roman-Reyna, Hagai Raanan, Gitta Coaker, Jonathan M. Jacobs, Doron Teper

**Affiliations:** 1 Dept. of Plant Pathology and Weed Research, Agricultural Research Organization—Volcani Institute, Rishon LeZion, Israel; 2 Dept. Of Plant Pathology and Environmental Microbiology, Pennsylvania State University, University Park, Pennsylvania, United States of America; 3 Dept. of Plant Pathology and Weed Research, Agricultural Research Organization—Gilat Research Center, Negev, Israel; 4 Dept. of Plant Pathology, University of California Davis, Davis, California, United States of America; 5 Dept. of Plant Pathology, The Ohio State University, Columbus, Ohio, United States of America; 6 Infectious Diseases Institute, The Ohio State University, Columbus, Ohio, United States of America; University of Cologne: Universitat zu Koln, GERMANY

## Abstract

Plant pathogenic bacteria often have a narrow host range, which can vary among different isolates within a population. Here, we investigated the host range of the tomato pathogen *Clavibacter michiganensis* (Cm). We determined the genome sequences of 40 tomato Cm isolates and screened them for pathogenicity on tomato and eggplant. Our screen revealed that out of the tested isolates, five were unable to cause disease on any of the hosts, 33 were exclusively pathogenic on tomato, and two were capable of infecting both tomato and eggplant. Through comparative genomic analyses, we identified that the five non-pathogenic isolates lacked the *chp/tomA* pathogenicity island, which has previously been associated with virulence in tomato. In addition, we found that the two eggplant-pathogenic isolates encode a unique allelic variant of the putative serine hydrolase *chpG* (*chpG*^C^), an effector that is recognized in eggplant. Introduction of *chpG*^C^ into a *chpG* inactivation mutant in the eggplant-non-pathogenic strain Cm^101^, failed to complement the mutant, which retained its ability to cause disease in eggplant and failed to elicit hypersensitive response (HR). Conversely, introduction of the *chpG* variant from Cm^101^ into an eggplant pathogenic Cm isolate (C48), eliminated its pathogenicity on eggplant, and enabled C48 to elicit HR. Our study demonstrates that allelic variation in the *chpG* effector gene is a key determinant of host range plasticity within Cm populations.

## Author summary

Most plant-pathogenic bacteria are specialists and have a narrow host range. However, the true host range of plant-pathogenic species is usually not well defined. Here, we demonstrate a variation in the host range of different clones of the specialist pathogen *Clavibacter michiganensis* (Cm), which is mainly reported to cause disease in tomato. We characterized a Cm clone library by combining dual-host virulence phenotyping, using tomato and eggplant, and genomic approaches. Our analysis showed that individual Cm clones harbor a differential host range. While most clones were solely pathogenic on tomato, a few were pathogenic on eggplant, and some were non-pathogenic in either host. Comparative genomic analyses identified that non-pathogenic clones lacked the *chp/tomA* pathogenicity island, a major virulence determinant of Cm. In addition, we found that eggplant-pathogenic clones harbored a distinct allelic variant of the putative secreted serine hydrolase *chpG*, a known immune elicitor in eggplant. We demonstrated that the presence of this allelic variant in eggplant-pathogenic Cm clones allows them to evade detection and cause disease. Our study provides novel insights into the phenotypic complexity within the population of bacterial plant pathogens and establishes a link between phenotypic variations and distinct genetic features.

## Introduction

Many bacterial plant pathogens are specialists by nature and found in association with a small number of hosts [1]. This phenomenon is reflected by the dichotomy of the broad host range of plant-associated bacteria such as *Xanthomonas* and *Pseudomonas syringae* on a genus or species level and the extremely narrow reported host range of pathovar or sub-species of the aforementioned groups [2,3]. The true host range of bacterial plant pathogens is usually not well defined, and, with the increased availability of sequencing technologies, both subspecies and pathovar classification and their reported host range are subjected to constant changes [4,5]. Compatibility with the unique physiology and genetic background of plant species or cultivar is one of the main features that determines whether a pathogen can utilize a plant as a host even when ecological factors are bypassed through artificial infection. Factors like the detoxification of plant-specific antimicrobials, the ability to degrade unique structural macromolecules, targeting host-specific susceptibility targets, and, avoidance or deactivation of host immune signaling have all been reported to play a key role in determining host specificity [6–8]. The most extensively studied host-determining factor in plant-pathogen interactions is the gene-for-gene type of immune recognition that confers recognition of pathogen effectors by host resistance protein [9]. Through this mechanism, a species or cultivar-specific immune receptor activates a strong immune response upon recognition of a unique secreted or translocated pathogen effector [10]. However, because of its elegant simplicity, gene-for-gene-based resistance can be relatively easily overcome by pathogens through modifications or removal of recognized effectors [11–13].

Bacterial canker caused by the actinobacteria *Clavibacter michiganensis* (Cm) is one of the most destructive bacterial diseases of tomato [14]. The disease is characterized by the appearance of stem cankers, wilting, ’bird’s eye’ fruit lesions, vascular collapse, and, in severe cases, death [14]. Cm enters the host through wounds and natural openings and extensively colonizes the host xylem vessels, spreading to all of the plant aerial tissues, including fruits and seeds [15,16]. Seed colonization is essential for the long-distance dispersal of Cm and its introduction into new habitats [17]. Through introduction by contaminated seeds, Cm has spread into most tomato-growing regions around the world and is considered an endemic pest in many countries, significantly affecting tomato production [14]. Because of the economic significance of the disease and the high risk of reintroduction via contaminated seeds, Cm populations were subjected to numerous phylogenetic analyses. These studies identified that the diversity within the Cm populations varies between countries. Cm populations in Turkey, Italy, and central Chile demonstrated low diversity, suggesting they mainly originated from founder populations that were established endemically in these countries [18–20]. On the other hand, high diversity was observed in Cm populations in Israel, New York State, Argentina, Iran, and Greece [21–25], suggesting multiple introductions of Cm, presumably through contaminated seeds.

Unlike many plant-pathogenic gram-negative bacteria, Cm does not possess a type III secretion system capable of delivering effectors directly into host cells. Instead, Cm pathogenicity heavily relies on secreted extracellular hydrolases and apoplastic effectors [26–29]. The main virulence determinants of Cm are encoded within three genomic regions: the pCM1 and pCM2 plasmids and the *chp/tomA* pathogenicity island (PAI), a 129 kb genomic island localized within the Cm chromosome [30]. pCM2 and the *chp/tomA* PAI encode for numerous putative secreted serine hydrolases that are classified into two main protein families, the Chp/Pat-1 putative serine proteases and the Ppa putative serine proteases. Both of these protein families play a crucial role in the virulence of Cm [31–33]. Homologs and paralogs within the Chp/Pat-1 family have been reported to contribute to aggression and colonization in Cm and other plant-pathogenic *Clavibacter* sp. such as *C*. *sepedonicus* and *C*. *capsici* [32–36]. However, their function and plant targets are unknown. In addition, serine protease activity has yet to be demonstrated in proteins of the Chp/Pat-1 family; this activity has only been inferred based on protein similarity and the observation that disruption of the serine hydrolase catalytic triad abolishes their ability to contribute to virulence or induce HR-like cell death in non-host plants [36–39].

Similar to other plant–pathogenic *Clavibacter* sp., Cm is a specialized pathogen that harbors a narrow host range. In field conditions, Cm is almost solely found in tomato, which serves as its main host [40], while infection in other Solanaceous crops such as potato, pepper, and eggplant is seldom reported [20,41]. The true host range of Cm is unclear and artificial infections of alternative hosts such as pepper and eggplant produce unstable and even contradictory results when conducted by different groups [42–44]. Recent studies by ourselves and Boyaci et al. identified that most domesticated eggplant varieties demonstrate moderate to high resistance to a number of Cm strains [45,46]. We identified that eggplant resistance to Cm is facilitated through immune recognition of ChpG, a secreted putative serine hydrolase of the Chp/Pat-1 family encoded by the *chp/tomA* PAI [46]. A *chpG* inactivation mutant in the background of the Cm model strain Cm^101^ cannot cause HR and is fully pathogenic in numerous eggplant varieties [46]. Introduction of *chpG* into the Cm^101^ *chpG* inactivation mutant restored immune recognition in eggplant, while introduction of a *chpG* variant containing a point mutation that results in the substitution of the serine residue within the predicted Ser/His/Asp catalytic triad at position 231 [33,39,46] to alanine failed to do the same [46]. This indicates that ChpG is a recognized effector, potentially through a byproduct of its putative catalytic activity, and therefore, its recognition in eggplant is likely to follow the gene-for-gene model. However, it is unclear whether this phenomenon is conserved in other clones within Cm populations.

In this study, we used functional and genomic analyses to determine the virulence and host range of 40 representative Cm isolates and identified that allelic variations in the *chpG* effector acts as a host range determinant.

## Results

### *Clavibacter michiganensis* isolates demonstrate variation in pathogenicity and host range

**Fig 1:**
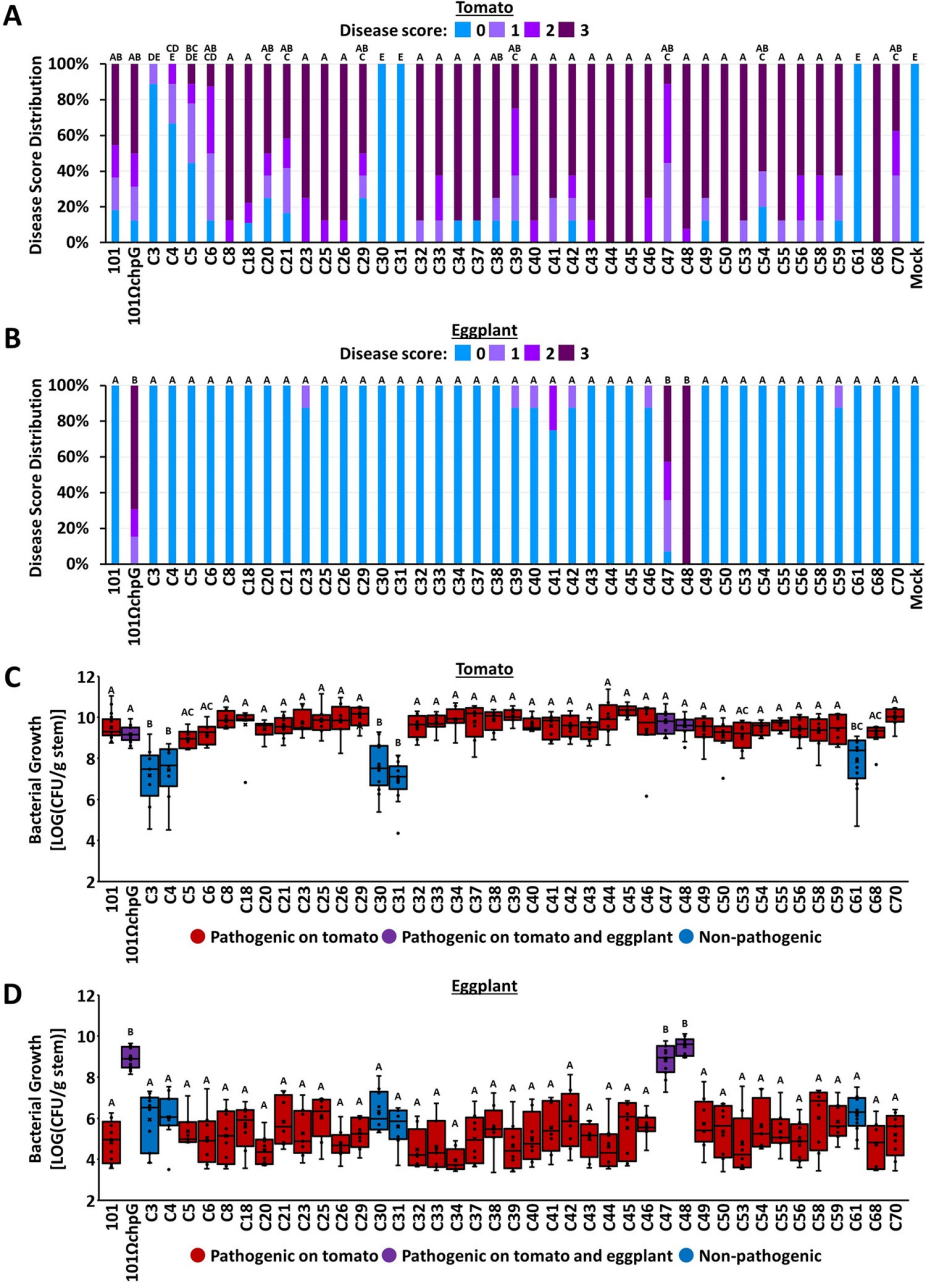
Symptoms and bacterial growth in plants inoculated with *Clavibacter michiganensis* (Cm) isolates. Four-leaf stage “Moneymaker” tomato plants (**A**, **C**) or three-leaf stage “Black Queen” eggplants (**B**, **D**) were inoculated with the indicated Cm isolates or water control (mock) by puncturing the stem area between the cotyledons with a wooden toothpick incubated in Cm solution (5 × 10^7^ CFU/ml). **(A**, **B**) Graphs represent the distribution of symptom severity in response to each isolate in at least eight plants taken from at least two experimental repeats. Symptoms severity were scored at 14 days post inoculations (dpi) according to the percentage of leaves displaying wilt (tomato) or leaf blotch (eggplant) symptoms by the following scale: 0 = no wilting/leaf blotch, 1 = 1–25%, 2 = 25–50%, 3 = 50–100%. **(C**, **D**) Stem bacterial populations 1 cm above the inoculation sites were quantified at 14 dpi. Lower and upper quartiles are marked at the margins of the boxes. Central lines, "×" and “o” represent medians, means and data points of at least eight biological repeats collected from at least two independent experiments. Boxes marked in red represent isolates that caused symptoms in tomato but not in eggplant, boxes marked in purple represent isolates that caused symptoms in tomato and in eggplant, and boxes marked in blue represent isolate that failed to cause symptoms in either tomato or eggplant. All depicted data were analyzed using one-way ANOVA followed by post-hoc Tukey HSD test. Letters indicate similarity in disease severity and bacterial populations (Tukey HSD test, *p value* ≤ 0.05).

Several studies from the past decade reported that a wide variety of *Clavibacter michiganensis* (Cm) isolates cannot cause disease in many eggplant accessions in controlled artificial inoculations [43,45,46]. These reports contradict the European and Mediterranean Plant Protection Organization (EPPO) Cm information page (EPPO code CORBMI) and earlier studies from the 1970s [43]. Considering that the majority of tested eggplant accessions demonstrated moderate to high resistance to Cm [46], we hypothesized that pathogenicity on eggplant is a unique feature of specific pathotypes within Cm populations. To test this, we screened a library of Cm isolates for virulence on eggplant and tomato. The Cm isolate library was composed of 40 isolates: 37 isolates were collected over a period of 29 years (from 1994 to 2023) from various regions in Israel and three additional reference clones originated from USA (C30 and C31) and the Netherlands (C20) (S1 Table). All isolates were tested for pathogenicity on tomato and eggplant by monitoring wilting (on tomato) or leaf blotch (on eggplant) symptoms and quantifying stem bacterial populations (Table 1, Figs 1, S1 and S2). We used the Cm model strains Cm^101^ and Cm^101^Ω*chpG* as a reference. Cm^101^Ω*chpG* is a *chpG* inactivation mutant in the background of Cm^101^ that we previously reported to be pathogenic on eggplant varieties [46]. 35 out of the 40 tested isolates were pathogenic on tomato, and were able to cause wilt symptoms and colonize tomato stems to approximately 10^8^–10^10^ CFU/gram tissue (Table 1, Figs 1 and S1). However, high variations were observed in the intensity of wilt symptoms and colonization capacity between the different pathogenic isolates (Fig 1). Five isolates (i.e. C3, C4, C30, C31, and C61) were non-pathogenic on tomato. These isolates failed to cause wilt symptoms and demonstrated a significant reduction in tomato stem colonization compared to the other clones (Table 1, Figs 1 and S1). In contrast to the tomato assays, 38 out of the 40 tested Cm isolates failed to cause leaf blotch symptoms on eggplant and were only able to colonize eggplant stems to approximately 10^4^–10^6^ CFU/gram tissue (Table 1, Figs 1 and S2). Two isolates, C47 and C48, were fully pathogenic on eggplant. These isolates caused leaf blotch symptoms and colonize eggplant stems 100–1000 fold higher than the other tested isolates, reaching approximately 10^8^–10^9^ CFU/gram tissue (Table 1, Figs 1 and S2).

**Table 1:** Virulence analyses of Cm isolates.

Isolate	Tomato symptoms^1^	Tomato growth^2^	Eggplant symptoms^1^	Eggplant growth^2^	Eggplant HR	*chpG* variant^3^	*chp/tomA* ^4^
Cm^101^	++	+++	-	+/-	+	A^S^	+
Cm^101^Ω*chpG*	++	+++	+++	+++	-	Ω^P^	+
C3	+/-	+	-	+/-	-	- ^G,P^	-
C4	+/-	+	-	+/-	-	- ^G,P^	-
C5	+	+++	-	+/-	+	B1^G,S^	+
C6	++	+++	-	+/-	+	D^G,S^	+
C8	+++	+++	-	+/-	+	B2^G^	+
C18	+++	+++	-	+/-	+	B1^G,S^	+
C20	++	+++	-	+/-	+	B1^G^	+
C21	++	+++	-	+/-	+	B1^G,S^	+
C23	+++	+++	+/-	+/-	+	B1^G^	+
C25	+++	+++	-	+/-	+	B1^G^	+
C26	+++	+++	-	+/-	+	B1^G^	+
C29	++	+++	-	+/-	+	B2^G,S^	+
C30	-	++	-	+/-	-	- ^G,P^	-
C31	-	+	-	+/-	-	- ^G,P^	-
C32	+++	+++	-	+/-	+	B1^G^	+
C33	+++	+++	-	+/-	+	B1^G^	+
C34	+++	+++	-	+/-	+	B1^G^	+
C37	+++	+++	-	+/-	+	B1^G^	+
C38	++	+++	-	+/-	+	B1^G^	+
C39	++	+++	+/-	+/-	+	B1^G^	+
C40	+++	+++	+/-	+/-	+	B1^G^	+
C41	+++	+++	+/-	+/-	+	B1^G^	+
C42	++	+++	+/-	+/-	+	B1^G^	+
C43	+++	+++	-	+/-	+	B1^G^	+
C44	+++	+++	-	+/-	+	B1^G^	+
C45	+++	+++	-	+/-	+	B1^G,S^	+
C46	+++	+++	+/-	+/-	+	B1^G,S^	+
C47	++	+++	++	+++	-	C^G,S^	+
C48	+++	+++	+++	+++	-	C^G,S^	+
C49	++	+++	-	+/-	+	B1^G^	+
C50	+++	+++	-	+/-	+	B1^G^	+
C53	+++	+++	-	+/-	+	B1^G^	+
C54	++	+++	-	+/-	+	B1^G^	+
C55	+++	+++	-	+/-	+	B1^G^	+
C56	+++	+++	-	+/-	+	B1^G^	+
C58	+++	+++	-	+/-	+	B2^G^	+
C59	++	+++	+/-	+/-	+	B2^G^	+
C61	-	++	-	+/-	-	-^G,P^	-
C68	+++	+++	-	+/-	+	B1^G,S^	+
C70	++	+++	-	+/-	+	B1^G^	+

^1^Average symptoms at 14 days post inoculation. (-) = no symptoms. (+/-) = symptom score between 0.1–1, (+) = symptom score between 1–1.5, (++) symptom score between 1.5–2.5, (+++) = symptom score between 2.5–3.

^2^Average bacterial growth (BG) [LOG (CFU/gram stem] at 14 days post inoculation. (+/-) = BG ≤ 6, (+) = 6 < BG ≤ 7.5, BG ≤ 6, (++) = 7.5 < BG ≤ 8.5, (+++) = 8.5 < BG.

^3^Allelic variant of *chpG* according to genome sequencing (G) and/or PCR (P) following Sanger sequencing (S).

^4^Presence of the *chp/tomA* genomic island according to genome sequencing.

**Fig 2:**
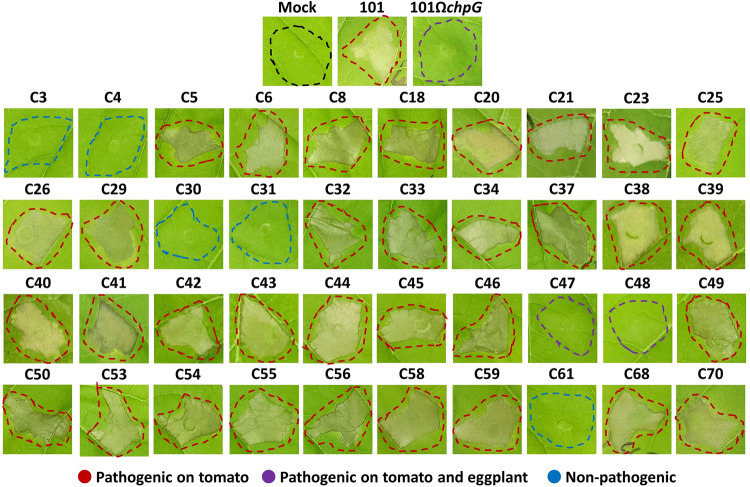
Elicitation of the hypersensitive response (HR) in eggplant leaves by *Clavibacter michiganensis* (Cm) isolates. Five to six leaf stage “Black Queen” eggplant leaves were infiltrated (10^8^ CFU/ml) with the indicated Cm isolates. Pictures were taken 48 h post infiltration. Infiltrated area are marked in colored dotted lines. Red lines represent isolates that caused symptoms in tomato but not in eggplant, purple lines represent isolates that caused symptoms in tomato and in eggplant, and blue lines represent isolate that failed to cause symptoms in either tomato or eggplant. Pictures are representatives of one out of at least 10 repeats from at least two independent experiments.

We previously reported that eggplant resistance to the Cm model strain Cm^101^ is accompanied by activation of hypersensitive response (HR) [46]. Hence, we monitored whether the Cm isolates in our library triggered HR in eggplant, and discovered that 33 isolates elicited HR in eggplant leaves. These include all tomato-pathogenic isolates with the exception of the two eggplant-pathogenic isolates C47 and C48 (Table 1, Fig 2). Surprisingly, all five tomato-non-pathogenic isolates (C3, C4, C30, C31, and C61) failed to elicit HR in eggplant as well, suggesting they lack a HR-inducing elicitor that is found in the tomato-pathogenic clones (Table 1, Fig 2). Our screen demonstrated that Cm pathogenicity on eggplant is isolate-dependent and associated with the inability to elicit HR on eggplant leaves.

### Genomic analyses of Israeli Cm isolates

**Fig 3:**
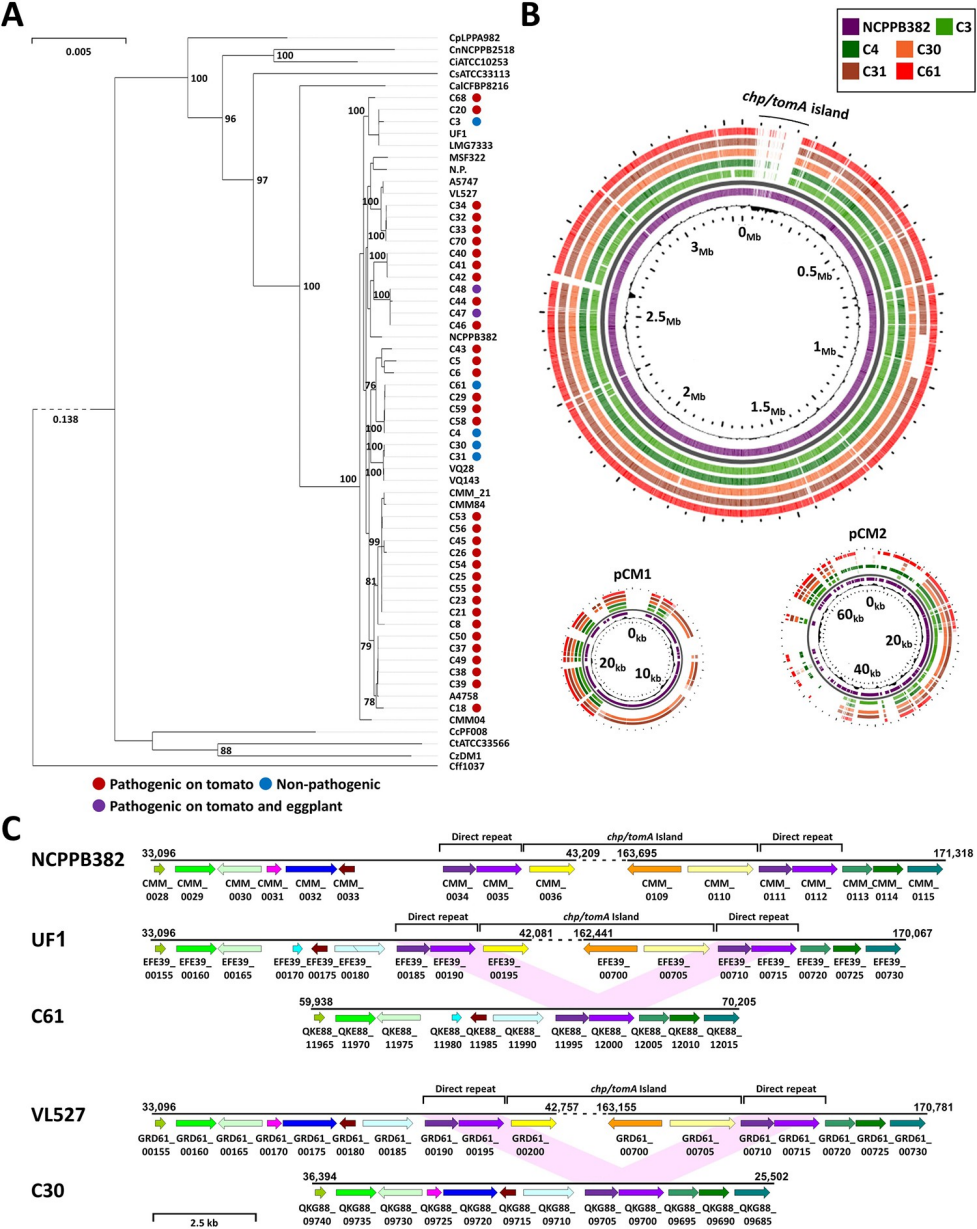
Tomato non-pathogenic *Clavibacter michiganensis* (Cm) isolates lack the *chp/tomA* pathogenicity island. (**A**) Phylogenetic tree of Cm isolates used in this study. The tree was produced using the M1CR0B1AL1Z3R web server (https://microbializer.tau.ac.il/) and is based on maximum-likelihood multiple sequence alignments of 129 core genes under default features and visualized by PhyD3. Genomes used for the analysis are 40 genomes of Cm isolates characterized in this study; references Cm strains UF1, LMG7333, NCPPB382, CMM84, Cmm_21, VQ143, VQ28, VL527, A5747, N.P., MSF322, and CMM04; and references strains of *C*. *sepedonicus* ATCC331133, *C*. *capsici* PF008, *C*. *nebraskensis* NCPPB2581, *C*. *insidiosus* ATCC10253, *C*. *tessellarius* ATCC33566, *C*. *zhangzhiyongii* DM1, *C*. *phaseoli* LPPA982, and *C*. *californiensis* CFBP8216. *Curtobacterium flaccumfaciens* Cff1037 was used as an outgroup. Isolates which were pathogenic on tomato but not pathogenic on eggplant are marked with a red dot, isolates which were pathogenic on tomato and eggplant are marked with a purple dot, and isolates which were non-pathogenic on tomato and eggplant are marked with a blue dot. (**B**) Whole genome alignment of the tomato non-pathogenic Cm isolates C3, C4, C30, C31, and C61 was performed against CDS of Cm strain NCPPB382 chromosome (NCBI GenBank: AM711867), pCM1 plasmid (AM711865) and pCM2 plasmid (AM711866) and visualized with BLAST atlas analysis in Gview server (https://server.gview.ca/) using default features. The *chp/tomA* genomic island (positions 42,081–162,441 in the NCPPB382 chromosome) is labeled. (**C**) Physical maps of the area surrounding the *chp/tomA* island in the tomato-pathogenic reference strains NCPPB382 (AM711867), UF1 (NZ_CP033724), and VL527 (NZ_CP047054) and representative corresponding regions in the tomato non-pathogenic isolates C61 [JASBOJ000000000, contig 16. Similar gene synteny was observed in C3 (JASBQC000000000, contig 13, 59,987–70,253) and C4 (JASBQB000000000, contig 14, 59,985–70,252)], and C30 [JASBPU000000000, contig 9. Similar gene synteny was observed in C31 (JASBPN000000000, contig 9, 36,376–25,484)]. Predicted ORFs are marked in arrows supplemented with locus tags in the corresponding genomes. Similar colors indicates the ORFs share DNA sequence identify of >97%, "/" indicates an ORF was disrupted by a frameshift mutation. The *chp/tomA* and direct repeat region are marked. The first ORF and the two last ORFs of the *chp/tomA* region are labeled in shades of yellow. The two ORFs encoded within the direct repeat region are labeled in shades of purple.

The Cm isolates used in our screen demonstrated high variation in their virulence and host range. To identify the source of these variations, we determined the genome sequences of the isolates in our library using the Illumina Nextseq2000 platform (BioProject ID PRJNA966807)(S2 Table). Next, we determined the phylogenetic lineage of the Cm isolates by conducting multiple sequence alignment of core genes (Fig 3A) using the M1CR0B1AL1Z3R web server [47]. The analysis was conducted along with NCBI genome deposits of Cm and non-Cm *Clavibacter* genomes, which were used as reference points. As expected, all 40 isolates clustered together with the NCBI Cm genome deposits (Fig 3A). These also include the eggplant-pathogenic and tomato non-pathogenic clones, indicating that virulence and host range variations exist within Cm populations. In addition, we observed multiple distinct phyletic clusters within the Israeli Cm isolates, some of which also included non-Israeli reference genomes. This observation supports previous fingerprinting-based phylogenetic analysis of Cm populations in Israel conducted in our institute [21,48](S1 Table) and suggests that Cm populations originated from multiple independent introductions that occurred throughout the years and not from a local founder population. While both eggplant-pathogenic isolates, C47 and C48, cluster together in the same lineage (Fig 3A, marked in purple), the five tomato-non-pathogenic isolates cluster into three independent lineages (Fig 3A, marked in blue), two of which contained tomato-pathogenic clones as well.

To assess the genetic differences between our isolates we conducted comparative genomic analysis by utilizing BLAST atlas feature in the Gview server [49]. All analyses were done in comparison to the Cm model strain NCPPB382 that served as a reference point. The comparative analysis identified that the *chp/tomA* pathogenicity island (PAI), which was reported to be the major chromosomal-encoded genomic region associated with virulence [30], was absent in all five tomato-non-pathogenic isolates (Fig 3B). In contrast, the *chp/tomA* PAI was present in all sequenced tomato-pathogenic isolates (S3 Fig). In addition, the presence of ORFs associated with the pCM1 and pCM2 plasmids demonstrated high variability between isolates (S3 Fig). Most plasmid variations were in genes that were reported to be required for plasmid maintenance [50,51] while genes associated with virulence such as *celA* and *pat-1* were conserved in most isolates (S3 Fig). This suggests that some isolates harbor plasmids with different replicons than that of pCM1 and pCM2 or potential genomic integration of the virulence-associated functions of the plasmids into the chromosome.

The absence of the *chp/tomA* PAI in the tomato non-pathogenic isolates can be an indicator that these isolates are either ancestral *Clavibacter* species that have yet to acquire the *chp/tomA* PAI or that the *chp/tomA* PAI was lost in the course of evolution. The scattered phyletic distribution of the tomato-non-pathogenic isolates and their phylogenetic clustering along with tomato-pathogenic isolates suggests a loss of the PAI. In further inquiry, we took a closer look at the surroundings of the region that the *chp/tomA* PAI was supposedly initially integrated into in both pathogenic and non-pathogenic isolates. As previously reported, in many pathogenic isolates, the *chp/tomA* regions were flanked by two ~1.9 kb direct repeats that share 98–99% DNA sequence identity [30,52]. These direct repeats match to positions 40,054–41,981 and 166,885–168,812 in the chromosome of Cm model strain NCPPB382 (Figs 3C and S4, marked in purple). Interestingly, all five tomato-non-pathogenic isolates harbored a single copy of this region that was surrounded in homologous areas to the upstream and downstream regions to the *chp/tomA* PAI in the pathogenic isolates (Figs 3C and S4). This suggest that the *chp/tomA* PAI was lost due to potential recombination between the direct repeats and that this event is more likely to occur independently in different isolates.

### Eggplant-pathogenic Cm isolates encode a unique allelic variant of the *chpG* effector

We previously reported that eggplant resistance to the Cm model strain Cm^101^ is mediated through HR-based immune recognition of the putative secreted serine hydrolase effector ChpG [46]. Therefore, we hypothesized that eggplant-pathogenic and HR-negative isolates are able to evade host recognition because they either lack a functional *chpG* homolog or encode for a *chpG* variant that is not recognized in eggplants. To examine this, we conducted *in silico* analysis for occurrence and polymorphisms in the *chpG* gene in our Cm isolate library. Our analysis identified five *chpG* allelic variants within Cm populations (Table 1) which we named *chpG*^A^, *chpG*^B1^, *chpG*^B2^,*chpG*^C^ and *chpG*^*D*^ (S5 and S6 Figs).

The *chpG*^A^ variant (CMM_0059) is found solely in the model strains NCPPB382 and Cm^101^. The *chpG*^B1^ variant (EFE39_00385) is the dominant variant in our library and the available NCBI Cm genome deposits (Table 1).The *chpG*^B2^ variant (LHJ47_00410) is found in four of the isolates from our library (Table 1) and the NCBI Cm genome deposits CMM39 and CMM04. *chpG*^C^ and *chpG*^D^ variants were unique to isolates in our library and were not identified in any of the NCBI Cm genome deposits. The *chpG*^C^ variant (QKF70_15430) was only present in the two eggplant-pathogenic isolates C47 and C48 while the *chpG*^D^ variant (QKG63_14905) was present in isolate C6 (Table 1).

**Fig 4:**
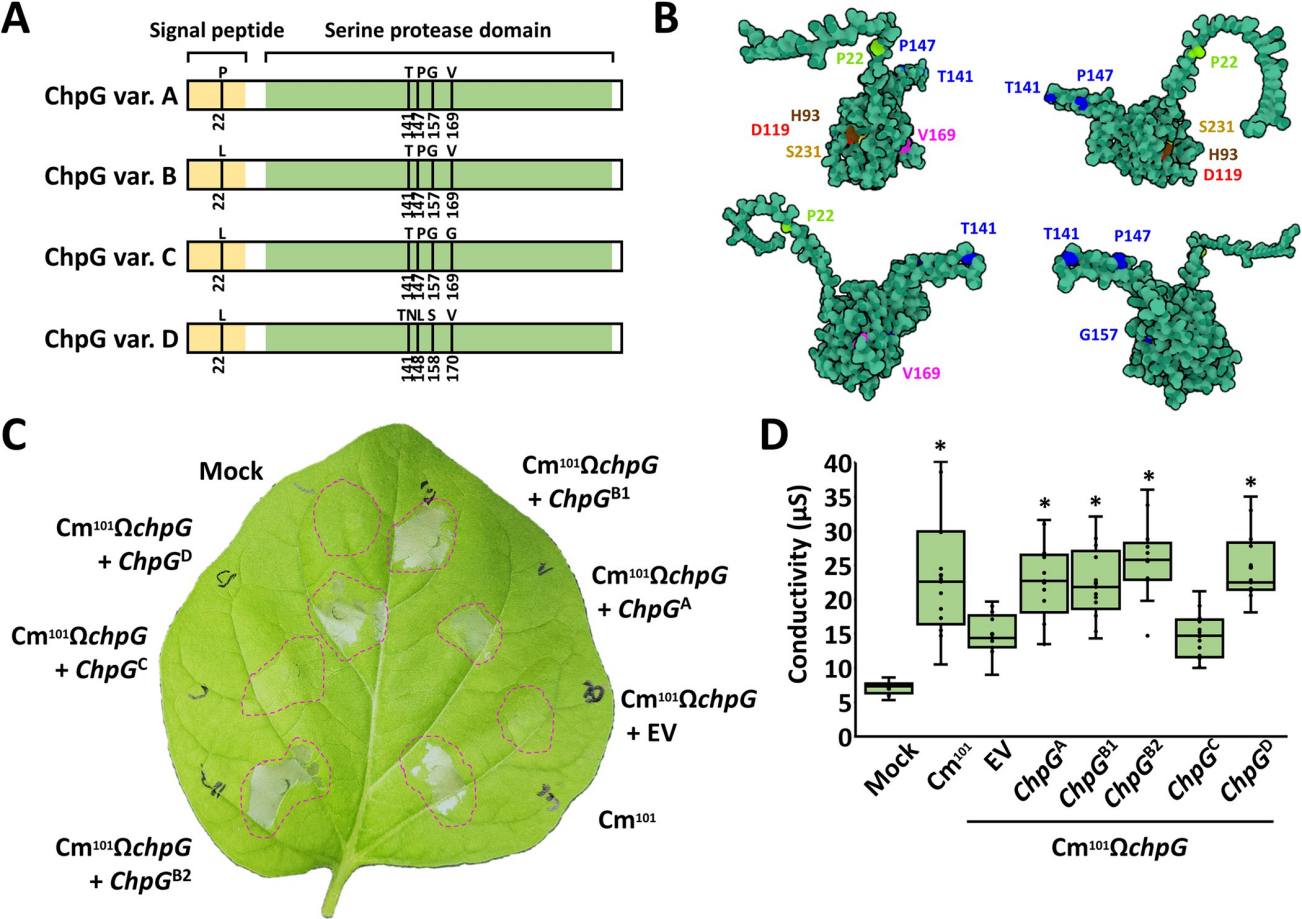
The ChpG allelic variants are differentially recognized in eggplant. **(A)** Schematic representation of the four ChpG protein variants depicted in S6 Fig. Signal peptide region, predicted by SignalP-5.0 (https://services.healthtech.dtu.dk/services/SignalP-5.0/) is labeled in yellow. Alpha-lytic serine protease domain, predicted by NCBI conserved domain search (https://www.ncbi.nlm.nih.gov/Structure/cdd/wrpsb.cgi) is labeled in green. Amino acid polymorphic sites are marked in black lines. **(B**) Model represents rotated forms of the predicted 3D structure of ChpG variant A (ChpG^A^). Structure prediction was conducted by Alphafold (https://alphafold.ebi.ac.uk/entry/A5CLZ4, UniPort num’ A5CLZ4) and visualized by Mol* (https://molstar.org/viewer/). Amino acid polymorphic sites unique to ChpG^A^, ChpG^C^ and ChpG^D^ are respectively marked in light green, magenta and blue. The H93, D119, and S231 amino acid residues representing the serine protease catalytic triad are respectively marked in brown, red and yellow. (**C, D**) Black Queen eggplant leaves were infiltrated with 10 mM MgCl_2_ (mock) or suspensions (10^8^ CFU/ml) of Cm^101^, and Cm^101^Ω*chpG* clones expressing the indicated *chpG* variants or empty vector control (EV). (**C**) Representative picture was taken 48 h post infiltration (hpi). (**D**) Cell death was quantified by ion leakage at 36 hpi. Lower and upper quartiles are marked at the margins of the boxes. Central lines and “o” represent medians and data points of 21 biological repeats collected from three independent experiments. "*" indicates significant differences (Mann–Whitney U test, *p-value* ≤ 0.05) from Cm^101^Ω*chpG* + EV.

We next examined how these allelic variations translate to differences in protein sequences (Figs 4A and S6) compared to the most abundant *chpG* allelic variant *chpG*^B1^. *chpG*^B2^ harbors a C78->T nucleotide substitution that results in a synonymous mutation and therefore did not affect amino acid composition, which was identical to that of *chpG*^B1^. We defined this protein variant as ChpG^B^. *chpG*^A^ has a single T65->C substitution that results in L22->P amino acid alteration. *chpG*^C^ has a single T506->G substitution which results in V169->G amino acid alteration. Finally, *chpG*^D^ harbors multiple nucleotide substitutions and insertion, which result in several amino acid alterations, including the insertion of Asn at amino acid position 142 and the amino acid substitutions of P147->L148 and G157->S158. Next, we mapped the amino acid modifications to the AlphaFold ChpG protein structure model (UniPort num’ A5CLZ4, Fig 4B)[53], which was visualized by Mol* viewer (https://molstar.org/viewer/). ChpG is predicted to have an unstructured N-terminal domain (positions 1–43) containing a sec-dependent signal peptide and a C-terminal serine protease domain composed of two beta-barrel domains which match the structure of serine proteases [54], and an external beta-sheet region (positions 126–155) that expend outside of the main serine protease domain (Figs 4A and 4B). Comparative structure analyses using 3D-BLAST [55](http://3d-blast.life.nctu.edu.tw/dbsas.php) and Foldseek [56](https://search.foldseek.com/search) identified that the ChpG structure shares the highest similarity to the S1 type secreted bacterial proteases Alpha-lytic serine protease of *Lysobacter enzymogenes* and Streptogrisin B of *Streptomyces griseus* [57–59].

We mapped the predicted amino acid polymorphic sites into the ChpG structure (Fig 4B). The ChpG^A^ polymorphic site at position 22 is placed within the unstructured N-terminal signal sequence, the ChpG^C^ polymorphic site at position 169 is located within the main conserved serine protease domain beta- barrel structure, while the three ChpG^D^ polymorphic sites are located within or in the proximity of the predicted external beta-sheet region (Fig 4B).

The *chpG*^C^ variant is uniquely encoded in the two eggplant-pathogenic strains C47 and C48, which suggests that this allelic variant is not recognized in eggplant. To test this, we monitored whether each one of the five *chpG* allelic variants can complement Cm^101^Ω*chpG* and restore recognition. All five *chpG* allelic variants were fused to a triple HA tag, cloned into the *E*. *coli*-*Clavibacter* shuttle vector pHN216 under the control of the constitutive *pCMP1* promoter and introduced into Cm^101^Ω*chpG*. Protein accumulation was confirmed in all transformants using western blot (S7A Fig). Eggplant leaves were infiltrated with cultures of Cm^101^Ω*chpG* carrying each of the allelic variants or empty vector control (EV) and monitored for HR for 48 h. HR was observed in Cm^101^Ω*chpG* carrying *chpG* variants A, B1, B2 and D while no HR was observed Cm^101^Ω*chpG* carrying *chpG* variant C (Fig 4C and 4D). Our data suggest that eggplant-pathogenic isolates harbor an adaptive modification in the *chpG* effector gene that abolishes its recognition in eggplant and enables these isolates to evade activation of host immune response.

### Purified ChpG^C^ variant does not elicit HR in eggplant

**Fig 5:**
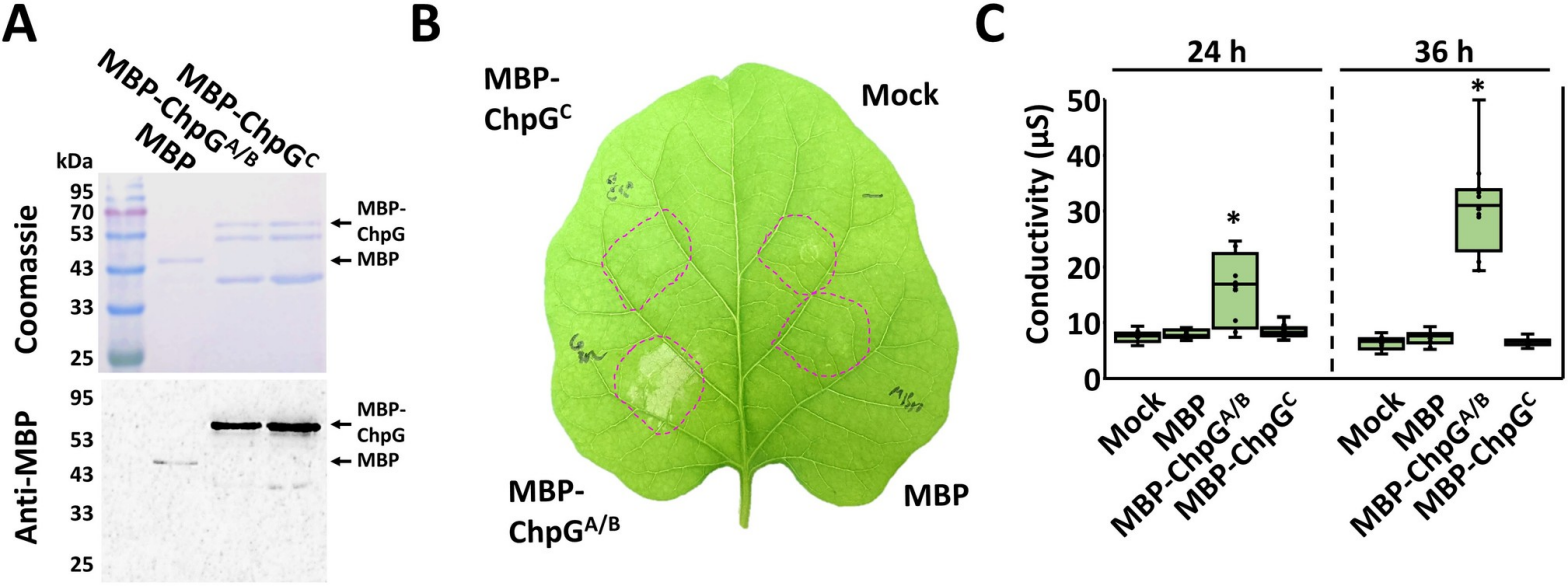
The ChpG^C^ variant does not elicit HR in eggplant. (**A**) Mature ChpG variants, lacking the predicted secretion sequence, were fused to a maltose-binding protein (MBP) tag, purified from *E*. *coli*, and visualized by SDS-PAGE using Coomassie blue staining (upper panel) and western blot analysis using anti MBP antibody (lower panel). **(B, C**) Purified proteins (0.01 μg/ml) or MgCl_2_ control (mock) were infiltrated into “Black Queen” eggplant leaves. **(B**) Representative leaf was photographed 36 hours post infiltration (hpi). (**C**) Cell death was quantified by ion leakage at 24 and 36 hpi. Lower and upper quartiles are marked at the margins of the boxes. Central lines and “o” represent medians and data points of at least 12 biological repeats collected from three independent experiments. "*" indicates a significant difference (Mann–Whitney U test, *p-value* ≤ 0.05) from mock control.

We previously showed that purified ChpG protein cloned from the eggplant-non-pathogenic Cm strain Cm^101^ elicit host-specific HR in eggplants [46]. We tested whether the ability to elicit HR is abolished in the purified ChpG^C^ variant, which failed to complement Cm^101^Ω*chpG*. The mature proteins of ChpG^A/B^ (represent both ChpG^A^ and ChpG^B^ since mature variants lack the predicted secretion signal) and ChpG^C^ were fused to maltose-binding-protein (MBP) tag, purified from *E*. *coli* (Fig 5A) and assayed for their ability to elicit HR in eggplant leaves upon syringe infiltration. The purified ChpG^A/B^ variant, which is found in eggplant-non-pathogenic isolates, elicited strong HR in eggplant leaves between 36–48 h post infiltration (hpi) (Fig 5B and 5C), while the purified ChpG^C^ variant failed to do the same (Fig 5B and 5C). Our analysis confirms that ChpG^C^ variant is not recognized in eggplant.

### Complementation analyses demonstrate differential recognition of *chpG* allelic variants

**Fig 6:**
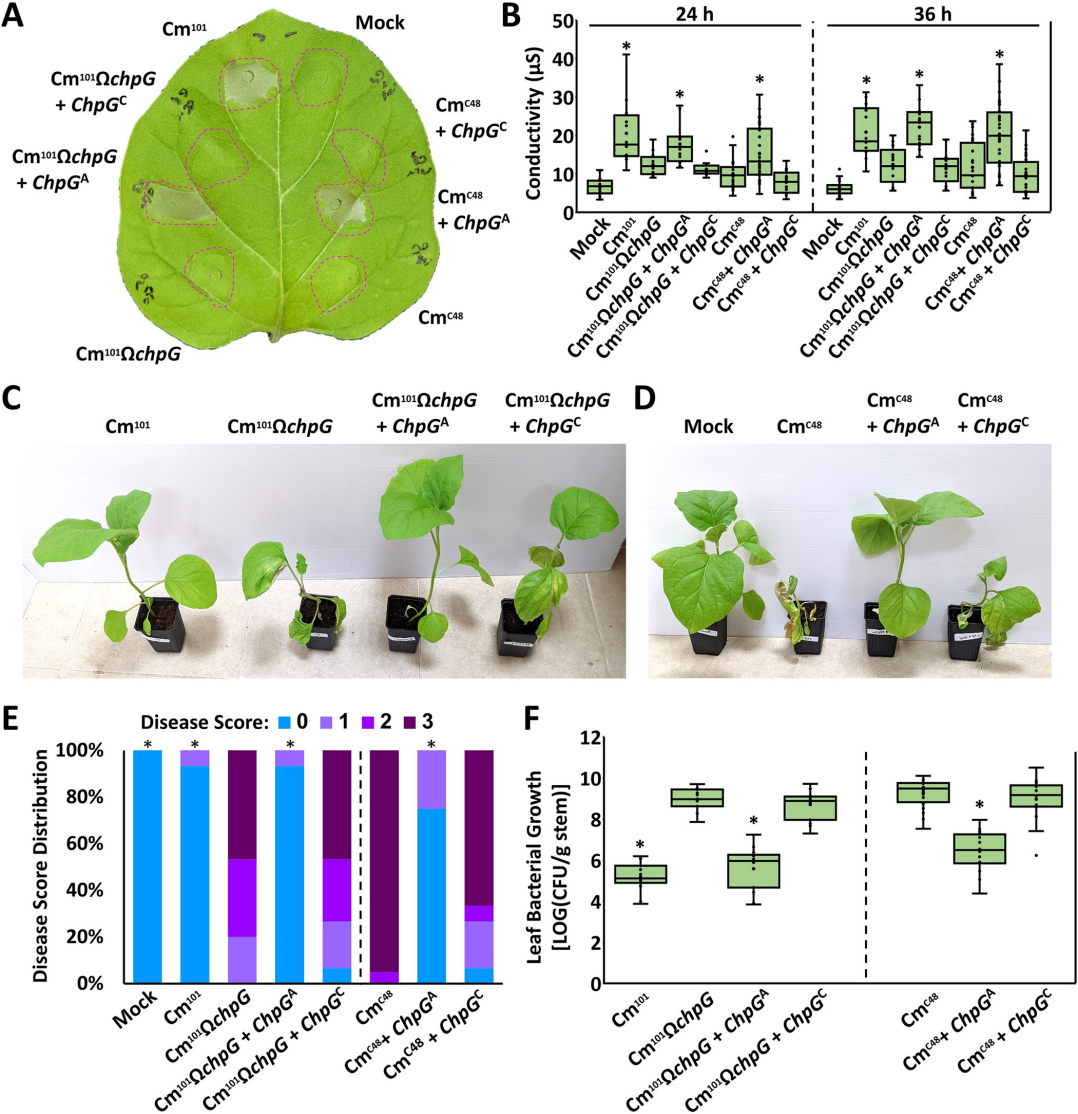
The ChpG^C^ variant does not restrict *Clavibacter michiganensis* (Cm) from colonizing eggplant. (**A, B**) Five to six leaf stage “Black Queen” eggplant leaves were infiltrated (10^8^ CFU/ml) with Cm^101^, Cm^101^Ω*chpG* and Cm^C48^ expressing the indicated *ChpG* variants or empty vector control (EV). (**A**) Picture was taken at 36 hours post inoculations (hpi). (**B**) Cell death was quantified by ion leakage at 24 and 36 hpi. Lower and upper quartiles are marked at the margins of the boxes. Central lines and “o” represent medians and data points of at least 12 (24 h) or 17 (36 h) biological repeats collected from at least two (24 h) or three (36 h) independent experiments. "*" indicates a significant difference (Mann–Whitney U test, *p-value* ≤ 0.05) from Cm^101^Ω*chpG* (left panel) or Cm^C48^ (right panel). (**C, D, E, F**) Three-leaf stage “Black Queen” eggplants were inoculated with the indicated Cm strains or water control (mock) by puncturing the stem area between the cotyledons with a wooden toothpick incubated in Cm solution (5 × 10^7^ CFU/ml). (**C, D**) Representative plants were photographed 14 days post inoculation (dpi). (**E**) Leaf blotch symptoms were quantified at 14 dpi according to the following scale: 0 = no leaf blotch, 1 = 1–25%, 2 = 25–50%, 3 = 50–100%. Graph depicts the symptom distribution in at least 15 plants pooled from at least three independent experiments. “*" indicates the score distribution is different from Cm^101^Ω*chpG* (left panel) or Cm^C48^ (right panel) (Pearson’s chi-squared test, *p-value* ≤ 0.05). (**F**) Stem bacterial populations 1 cm above the inoculation sites were quantified at 14 dpi. Lower and upper quartiles are marked at the margins of the boxes. Central lines and “o” represent medians and data points of 15 biological repeats collected from at least three independent experiments. "*" indicates a significant difference (Mann–Whitney U test, *p-value* ≤ 0.05) from Cm^101^Ω*chpG* (left panel) or Cm^C48^ (right panel).

To prove that the occurrence of different allelic variants of *chpG* indeed determines the host range of Cm isolates, we conducted reciprocal complementation analyses. For that, pHN216 carrying *chpG*^A^ and *chpG*^C^ were introduced into the eggplant pathogenic isolates C48 (Cm^C48^) and Cm^101^Ω*chpG*, and transformants were assayed from HR elicitation and virulence in eggplant. Protein accumulation of ChpG^A^ and ChpG^C^ was monitored in the two strains and demonstrated that while both ChpG variants were present in premature and mature forms, ChpG^A^ accumulation was significantly higher in both Cm^C48^ and Cm^101^Ω*chpG* compared to ChpG^C^, suggesting that allelic polymorphism in *chpG* might affect its translation, secretion efficiency, or protein stability (S7A, S7B and S7C Fig). Cm^C48^ and Cm^101^Ω*chpG* carrying pHN216:*chpG*^A^ were able to elicit HR upon infiltration into eggplant leaves between 24–48 hpi, while Cm^C48^ and Cm^101^Ω*chpG* carrying pHN216:*chpG*^C^ or pHN216 empty vector control failed to do the same (Fig 6A and 6B). Virulence assays were conducted using stab inoculations of eggplant stems. Virulence was quantified by monitoring leaf blotch symptoms and stem bacterial populations two weeks post inoculations. As previously reported, C48 and Cm^101^Ω*chpG* caused significant leaf blotch symptoms on eggplant (Fig 6C, 6D and 6E). However, introduction of pHN216:*chpG*^A^ but not pHN216:*chpG*^C^ turned both Cm isolates to non-pathogenic on eggplant (Fig 6C, 6D and 6E). Stem bacterial populations of Cm^C48^ and Cm^101^Ω*chpG* carrying pHN216:*chpG*^C^ were similar to Cm^C48^ and Cm^101^Ω*chpG* and reached approximately 10^9^ CFU/gram stem (Fig 6F). In contrast, bacterial population of Cm^C48^ and Cm^101^Ω*chpG* carrying pHN216:*chpG*^A^ demonstrated a 200- to 1000-fold reduction compared to their parental clones and reached approximately 10^6^–5 × 10^6^ CFU/gram stem (Fig 6F). To eliminate the possibility that introduction of pHN216:*chpG*^A^ or pHN216:*chpG*^C^ resulted in a non-host-specific alteration in virulence, Cm^C48^ and Cm^101^Ω*chpG* transformed clones were assayed for virulence on tomato and demonstrated no significant changes in their ability to cause disease (S8 Fig). Our data shows that the *chpG*^A^ significantly hinders the virulence of Cm^C48^ and Cm^101^Ω*chpG* on eggplant while *chpG*^C^ does not.

### A single amino acid substitution in the ChpG serine protease domain eliminates its recognition in eggplant

**Fig 7:**
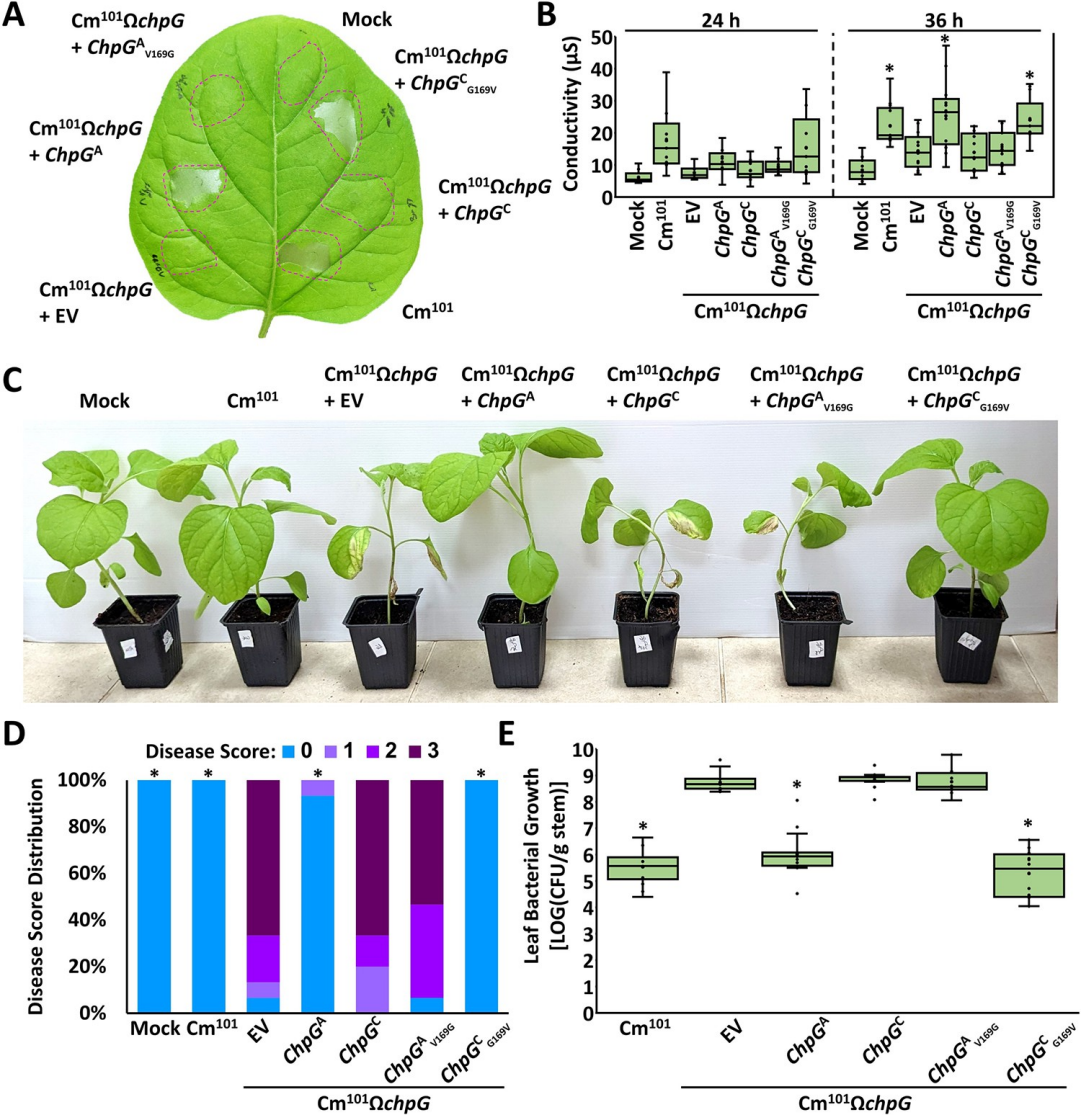
Differential recognition of ChpG variants is linked to a single polymorphic amino acid. (**A, B**) Five to six leaf stage “Black Queen” eggplant leaves were infiltrated (10^8^ CFU/ml) with Cm^101^, Cm^101^Ω*chpG* expressing the indicated *ChpG* variants or empty vector control (EV). (**A**) Picture was taken at 36 hours post inoculation (hpi). (**B**) Cell death was quantified by ion leakage at 24 and 36 hpi. Lower and upper quartiles are marked at the margins of the boxes. Central lines and “o” represent medians and data points of at least 9 (24 h) or 11 (36 h) biological repeats collected from two independent experiments. "*" indicates significant difference (Mann–Whitney U test, *p-value* ≤ 0.05) from Cm^101^Ω*chpG*. (**C, D, E**) Three-leaf stage “Black Queen” eggplants were inoculated with the indicated Cm strains or water control (mock) by puncturing the stem area between the cotyledons with a wooden toothpick incubated in Cm solution (5 × 10^7^ CFU/ml). (**C**) Representative plants were photographed 14 days post inoculations (dpi). (**D**) Leaf blotch symptoms were quantified at 14 dpi according to the following scale: 0 = no leaf blotch, 1 = 1–25%, 2 = 25–50%, 3 = 50–100%. Graph depicts the symptom distribution in at least 15 plants pooled from three independent experiments. “*" indicates the score distribution is different from Cm^101^Ω*chpG* (Pearson’s chi-squared test, *p-value* ≤ 0.05). (**E**) Stem bacterial populations 1 cm above the inoculation sites were quantified at 14 dpi. Lower and upper quartiles are marked at the margins of the boxes. Central lines and “o” represent medians and data points of 16 biological repeats collected from three independent experiments. "*" indicates a significant difference (Mann–Whitney U test, *p-value* ≤ 0.05) from Cm^101^Ω*chpG*.

ChpG^A^ and ChpG^C^ differ from each other due to two amino acid substitutions, at position 22 which is predicted to be part of the signal peptide and at position 169 that is part of the serine protease domain (Fig 4A). Purified mature ChpG^A/B^, which lacks the signal peptide region, elicits HR in eggplant leaves (Fig 5B and 5C), suggesting that valine to glycine alteration at position 169 in ChpG^C^ allows it to evade recognition in eggplant. To test this, we conducted reciprocal substitutions of amino acid position 169 in ChpG^A^ and ChpG^C^. Val169 in ChpG^A^ was substituted to Gly (ChpG^A^_V169G_), while Gly169 in ChpG^C^ was substituted to Val (ChpG^C^_G169V_). We note that Leucine is found at position 22 in both ChpG^B^ and ChpG^C^ and therefore the amino acid sequence of ChpG^C^_G169V_ is identical to ChpG^B^. pHN216-based plasmids carrying ChpG^A^_V169G_ or ChpG^C^_G169V_ fused to HA tag were introduced into Cm^101^Ω*chpG*. We monitored protein accumulation by western blot and observed that amino acid substitutions in position 169 did not affect the accumulation of ChpG in mature or premature forms (S7C Fig). This indicates that the observed differences in protein accumulation between ChpG^A^ and ChpG^C^ (S7A, S7B and S7C Fig) is likely to be linked to the signal sequence polymorphic site at position 22 and not to the polymorphic site at position 169. After confirming protein expression, the transformed Cm^101^Ω*chpG* clones were monitored for their ability to elicit HR, cause disease and colonize eggplant. As expected, the V169G substitution in ChpG^A^ abolished its ability to complement Cm^101^Ω*chpG* and bacteria failed to elicit HR and were as pathogenic in eggplant as Cm^101^Ω*chpG* carrying *chpG*^C^ or pHN216 empty vector control (EV) (Fig 7). Correspondingly, the G169V substitution in ChpG^C^ enable it to complement Cm^101^Ω*chpG* and bacteria elicited HR and lost their pathogenicity on eggplant in a similar manner to Cm^101^Ω*chpG* carrying *chpG*^A^ or Cm^101^ (Fig 7). Our analysis confirmed that a single amino acid alteration that occurred in the serine protease domain of ChpG^C^ enables it to evade recognition in eggplant.

### ChpG^C^ elicits attenuated HR in the non-host plant *Mirabilis jalapa*

**Fig 8:**
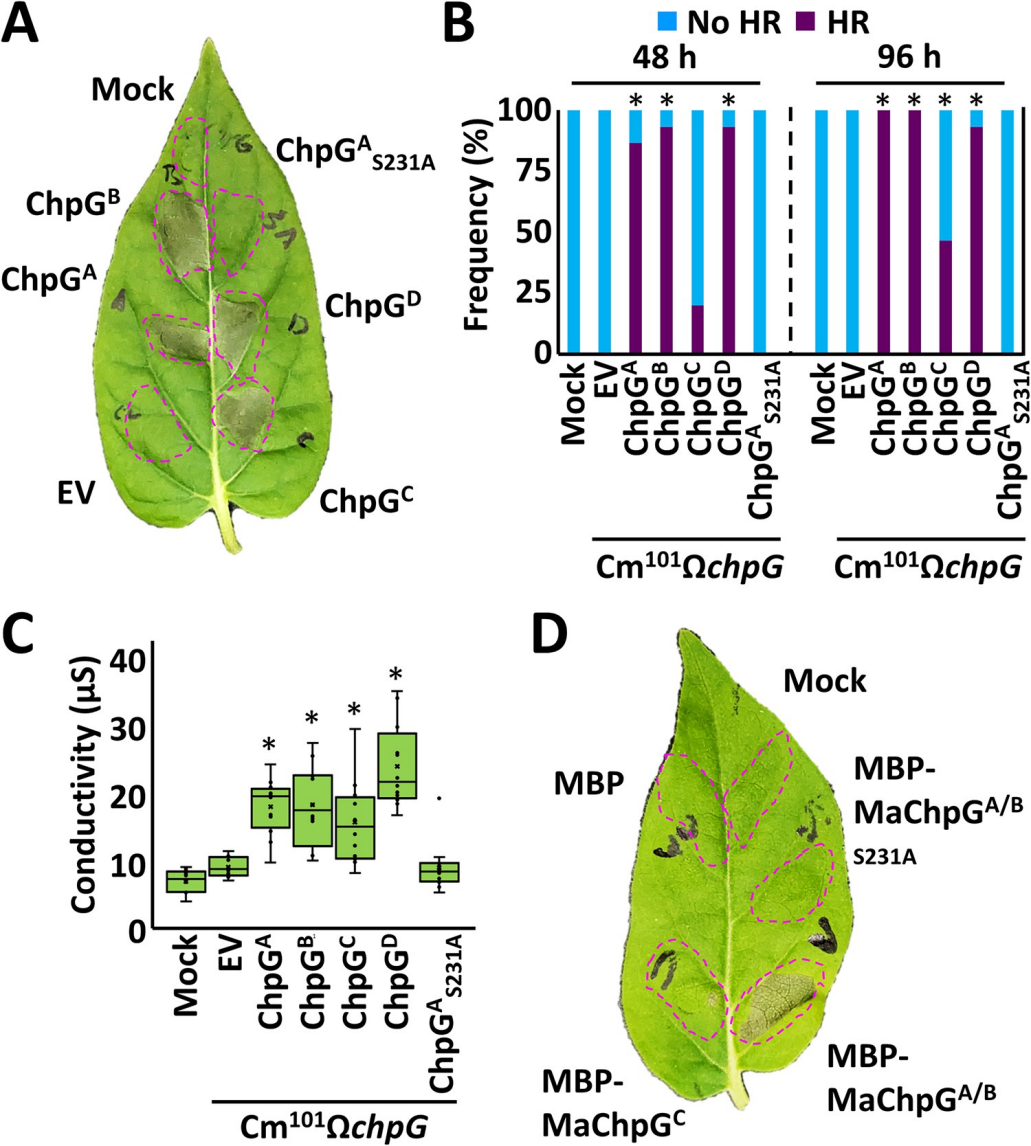
Immune recognition of ChpG variants in *Mirabilis jalapa*. (**A, B, C**) Eight to ten leaf stage *Mirabilis jalapa* leaves were infiltrated (10^8^ CFU/ml) with Cm^101^Ω*chpG* expressing the indicated *ChpG* variants or empty vector control (EV). (**A**) Representative leaf was photographed at 96 hours post inoculations (hpi). (**B**) HR was monitored at 48 and 96 hpi in the infiltrated areas and each infiltration point received a HR classification (HR or no HR). HR frequencies of 15 biological repeats collected from three independent experiments are presented in a stacked bar graph. “*" indicates the score distribution is different from Cm^101^Ω*chpG* + EV (Pearson’s chi-squared test, *p-value* ≤ 0.05). (**C**) Cell death was quantified by ion leakage at 96 hpi. Lower and upper quartiles are marked at the margins of the boxes. Central lines and “o” represent medians and data points of at least 12 biological repeats collected from two independent experiments. "*" indicates a significant difference (Mann–Whitney U test, *p-value* ≤ 0.05) from Cm^101^Ω*chpG* + EV. (**D**) *Mirabilis jalapa* leaves were infiltrated with the indicted MBP fused ChpG variants (0.01 μg/ml) or MgCl_2_ (mock). Representative leaf was photographed 3 days later. The image represents 18 biological replicates across three independent experiments, all showcasing comparable results.

In addition to eggplant, ChpG has been reported to induce HR in the non-host plant *Mirabilis jalapa* [33]. We assessed whether the evasion of immune recognition by the ChpG^C^ variant is unique to eggplant or if it can be extended to different hosts as well. To that aim, *Mirabilis jalapa* leaves were infiltrated with Cm^101^Ω*chpG* strains introduced with either of the four natural *chpG* variants (*chpG*^A^, *chpG*^B^, *chpG*^C^, and *chpG*^D^), a putative catalytically inactive ChpG^A^ variant, *chpG*^A^_S231A_ [46], and empty vector control (EV) and monitored for HR for four days. HR was induced by Cm^101^Ω*chpG* strains carrying *chpG*^A^, *chpG*^B^, *chpG*^C^, or *chpG*^D^ but not Cm^101^Ω*chpG* strains carrying EV or *chpG*^A^_S231A_, indicating that putative catalytic activity of ChpG is likely to be required for its recognition in *Mirabilis jalapa* (Fig 8A and 8B). However, we observed clear differences in the kinetics and frequency of HR induced by *chpG*^C^ compared to the other variants. Cm^101^Ω*chpG* strains carrying *chpG*^B^, *chpG*^C^, or *chpG*^D^ typically induced HR within 24–48 h in most leaves while HR induced by Cm^101^Ω*chpG* carrying *chpG*^C^ was typically induced within 72–96 h in 40%-50% of the infiltrated leaves (Fig 8B and 8C), suggesting that ChpG^C^ is not recognized as effectively in *Mirabilis jalapa* compared to the other variants. To further support this data, we monitored HR in *Mirabilis jalapa* infiltrated with purified MBP-fused ChpG^A/B^, ChpG^C^, and ChpG^A/B^_S231A_ and identified HR was only induced by MBP-ChpG^A/B^ but not by MBP-ChpG^C^, MBP-ChpG^A/B^_S231A_ and MBP control (Fig 8D).

The experiments suggest that ChpG-mediated HR in the non-host plant *Mirabilis jalapa* relies on its putative catalytic activity and demonstrate that the ChpG^C^ variant causes significantly attenuated HR in this host.

### Comparative structural modeling and serine hydrolase activity assays of ChpG variants

ChpG-mediated HR is abolished upon substitution of the serine within the serine hydrolase catalytic triad with alanine in two phylogenetically distant hosts, eggplant and *Mirabilis jalapa*, indicating that potential enzymatic activity is required for its immune recognition [46]. Therefore, it is possible that the V169G alteration in the ChpG^C^ variant alters or abolishes enzymatic activity or changes substrate specificity. The location of V169 within the serine protease domain (Fig 4B) suggests that this might be the case. However, amino acid alignment of Chp/Pat-1 homologs did not find V169 to be conserved within the protein family [33]. To estimate whether V169G has a significant effect on protein fold, we predicted the 3D structure of ChpG^A^ and ChpG^A^_V169G_ using the alphafold2 modeling platform. Structural alignment of the predicted structures demonstrated only minor localized angle shift in the alpha-helix structure in the proximity of the altered residue while structure alignment was unaltered throughout the rest of the protein (S9A Fig). However, molecular dynamic simulation of the predicted structures of ChpG^A^ and ChpG^A^_V169G_ imply that the V169G substitution results in a decrease in the structural stability of ChpG^A^_V169G_. The Root Mean Square Fluctuation (RMSF, which represents the level fluctuation of individual residues from their average positions over a simulation trajectory) of ChpG^A^_V169G_ shows a destabilized region between residues 120 to 170 compared to ChpG^A^ (S9B Fig).

**Fig 9:**
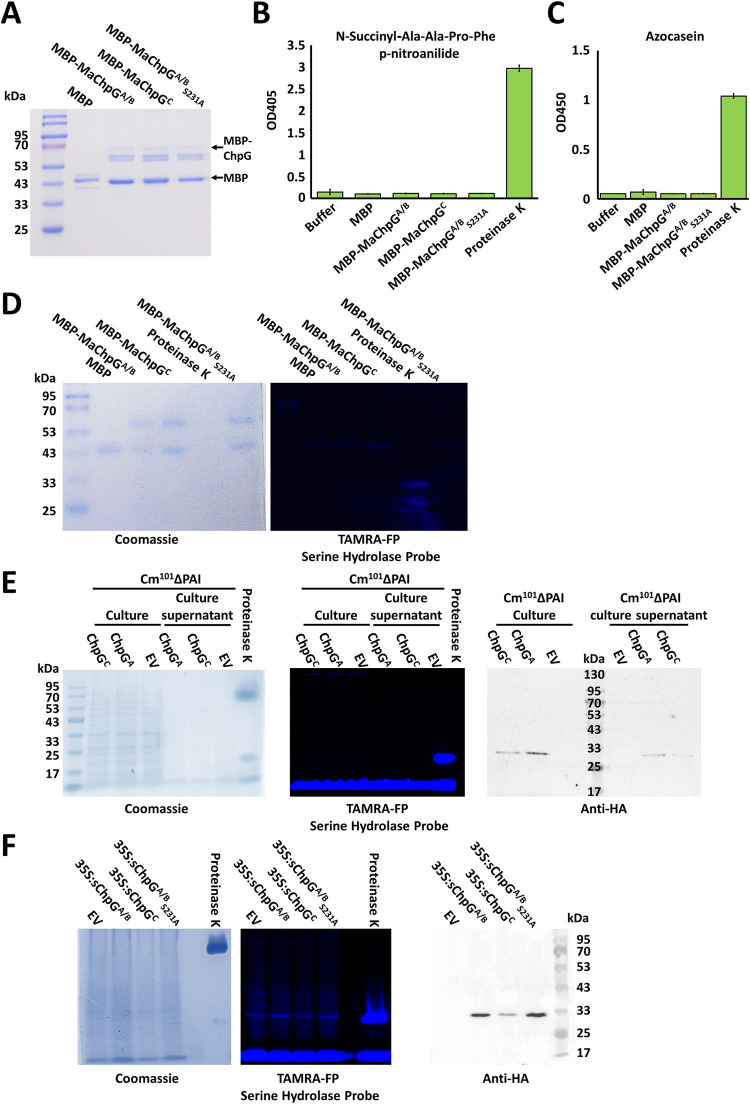
Serine hydrolase activity assays in ChpG variants failed to detect enzymatic activity. (**A**) Mature ChpG variants, lacking the predicted secretion sequence, were fused to a maltose-binding protein (MBP) tag, purified from *E*. *coli*, and visualized by SDS-PAGE using Coomassie blue staining. (**B**) N-Succinyl-Ala-Ala-Pro-Phe p-nitroanilide cleavage assays were conducted using 1 μg of the indicated MBP fusion proteins or 0.1 μg proteinase K. Data represents the averages and standard divisions of three technical repeats. (**C**) Azocasein hydrolysis assays were conducted using 1 μg of the indicated MBP fusion proteins or 0.1 μg proteinase K. Data represents the averages and standard divisions of at least four technical repeats. (**D, E, F**) Serine hydrolase activity using the TAMRA-FP serine hydrolase probe was assessed with 0.1 (**D**) or 1 (**E**, **F**) μg proteinase K (positive control), 1 μg of the indicated MBP fusion proteins (**D**), total protein extracts, and supernatants of Cm^101^ΔPAI cultures introduced with pHN216-based plasmids expressing the indicated *chpG* variants under the *pCMP1* promoter fused to an HA tag or an empty vector control (EV) (**E**), or total protein extracts from *Nicotiana benthamiana* leaves infiltrated with *Agrobacterium* carrying vectors aimed at transient expression of the indicated mature *chpG* variants fused to the 33 aa secretion signal of NtPR1 at the N-terminus and to an HA tag at the C-terminus under the control of the *CaMV35S* promoter or the 33 aa secretion signal of NtPR1 alone (EV) (**F**). **Left panels** (**D, E, F)**: Total protein was visualized by Coomassie blue staining. **Middle panels** (**D, E, F)**: Active site fluorescent binding was visualized by fluorescent imaging (530 nm excitation, 554 nm emission). **Right panels** (**E**, **F**): Confirmation of expression of the ChpG variants by western blot using anti-HA antibody. Experiments were repeated Twice (**B**, **C**) or three times (**D, E, F**) with similar results.

To address whether the predicted structural instability affects protein function, we conducted *in vitro* peptidase activity assays with purified ChpG^A^, ChpG^C^, and the putative catalytically inactive ChpG^A^ variant ChpG^A^_S231A_, using the colorimetric chymotrypsin substrate N-Succinyl-Ala-Ala-Pro-Phe p-nitroanilide and the nonspecific protease substrate Azocasein. However, we failed to detect peptidase activity in any of the ChpG variants (Fig 9A, 9B and 9C).

The inability of the ChpG variants to hydrolyze the two substrates can be a result of multiple factors, such as serine hydrolase substrate specificity, reduced activity due to expression in a heterologous system, or dependency on activation in the plant apoplast through modification by a plant factor or by the apoplast environment itself. To address these issues, we conducted a series of serine hydrolase assays using a serine hydrolase active-site directed probe, which has been demonstrated to act on diverse families of serine hydrolases and serves as a reporter for serine hydrolase activity and activation in bacterial, plant, and animal systems [60–63]. Active site binding assays were conducted using the ChpG^A^, ChpG^C^, and ChpG^A^_S231A_ variants, which were purified from *E*. *coli*, overexpressed in Cm, or transiently overexpressed in *Nicotiana benthamiana* using *Agrobacterium* (Fig 9D, 9E, and 9F). To monitor serine hydrolase activity in ChpG-overexpression Cm clones, pHN216 carrying *chpG* variants expressed under the control of the *pCMP1* promoter or an empty vector control were introduced into Cm_101_ ΔPAI (CMM30-18)[30], which lacks the *chp/tomA* PAI and the pCM2 plasmid and, therefore, does not encode any homologs of the Chp/Pat-1 family. Serine hydrolase activity was assessed in culture lysates and culture supernatants. To monitor serine hydrolase activity *in planta*, mature ChpG^A^, ChpG^C^ and ChpG^A^_S231A_ variants were translationally fused to the N-terminal signal peptide of NtPR1a (ssNtPR1a), transiently expressed in *Nicotiana benthamiana* under the control of *CaMV 35S* promoter via *Agrobacterium*, and serine hydrolase activity was assessed in total protein extract. Expression of the *chpG* variants in Cm cultures and *N*. *benthamiana* leaves was validated by western blot (Fig 9E and 9F). In addition, we confirmed that the expression systems did not affect the ability of ChpG^A^ to elicit HR, which was evident in eggplant leaf areas infiltrated with Cm^101^ΔPAI + *chpG*^A^ and *Agrobacterium* carrying a binary vector for the expression of *chpG*^A^, but not in those with the empty vector or the other *chpG* variants, nor in *N*. *benthamiana* infiltrated areas expressing *chpG*^A^ (S10 Fig). While serine hydrolase activity was clearly visible with proteinase K, which served as a positive control, we failed to detect differential serine hydrolase activity between the ChpG variants and the purified MBP protein or the empty vector controls in any of the three experimental systems (Fig 9D, 9E, and 9F).

## Discussion

Plant pathogenic bacteria are subjected to high selective pressures to adapt to their host plants and environment [64]. These pressures are potentially increased in seed-borne pathogens that, in addition to constant competition from newly introduced haplotypes, need to adjust to local niches [65]. This will dynamically affect local population structures and encourage specialization and establishment of regional adaptive haplotypes that potentially possess different traits than the founder populations [66]. Such localized adaptations may challenge the preexisting knowledge of pathogen host range, epidemiology, and pathogenesis mechanisms, which is usually extrapolated from studies that were conducted with representative model strains. In this study, we conducted an in-depth characterization of the Cm population in Israel that combined genomics and virulence-based phenotypic assays. In addition to tomato, we used eggplant in our virulence assays to address the discrepancy in reports regarding whether it is a host of Cm [42,43,45,46]. We recently published that eggplant resistance to the Cm model strain Cm^101^ is facilitated through immune recognition of the *chp/tomA* PAI-encoded secreted putative serine hydrolase ChpG [46]. Since most available Cm genomes encode a *chpG* homolog, we hypothesized that eggplant pathogenicity is unique to a Cm subpopulation that is able to evade ChpG recognition. Our findings support this hypothesis and show that while most Cm isolates elicit HR, and are not pathogenic on eggplant; this is not the case for two isolates, C47 and C48. C47 and C48 harbor a unique allelic variant of *chpG* (*chpG*^C^) that is not recognized in eggplant, enabling disease development. This allelic variant differs from the other four *chpG* allelic variants in the Cm population by a single SNP that results in a V169G substitution located in the serine protease beta-barrel domain. Intriguingly, we did not find the *chpG*^C^ variant in any of the Cm genome deposits available in NCBI, suggesting that this variant is unique to the Israeli Cm population. This is supported by the high phylogenetic proximity between C47 and C48 to other Israeli Cm isolates such as C44 and C46, which do not harbor the V169G substitution (Fig 3A). Therefore, it is more likely that C47 and C48 originated from a parental clone within the Israeli population that acquired this adaptive mutation.

It is unclear how the V169G substitution disrupts the recognition of ChpG in eggplant. Considering that substitution of the conserved serine in the serine hydrolase catalytic triad to alanine abolishes the recognition of ChpG in eggplant [46], it is possible that ChpG^C^ variant is inactive, has reduced hydrolase activity, or exhibits alterations in the tertiary structure affecting recognition. We attempted to address this issue by monitoring *in vitro* and *in vivo* serine hydrolase activity but failed to identify activity by either ChpG^C^ or the eggplant HR-inducing variant ChpG^A^. We note that while the virulence contribution and non-host induction of HR have been well documented for putative proteases of the Chp/Pat-1 family [34–36,46], their host targets, molecular function, and activation mode have yet to be characterized, and neither *in vitro* nor *in planta* serine hydrolase activity has been demonstrated to date. This information gap is not unique to Chp/Pat-1 putative proteases. In contrast to mammalian pathogens such as *Staphylococcus aureus* and *Streptococcus pneumonia* [67,68], extracellular virulence-associated proteases of plant pathogenic bacteria are seldom subjected to in-depth biochemical and functional studies, despite their importance to virulence in numerous pathogens such as *Clavibacter*, *Pectobacterium*, and *Burkholderia glumae* [69–72]. Nevertheless, serine hydrolase activity of Chp/Pat-1 has been indirectly supported through the loss of virulence and/or HR-inducing function *in planta* by amino acid substitution of the Ser-His-Asp catalytic triad and other conserved residues within the catalytic domain [34–36,38,46], suggesting that Chp/Pat-1 are likely to be enzymatically active [69]. Interestingly, HR elicitation by ChpG^C^ in the non-host plant *Mirabilis jalapa* is significantly reduced compared to other eggplant HR-inducing ChpG variants. Considering that ChpG recognition in both eggplant and *Mirabilis jalapa* relies on an intact catalytic triad, it is plausible that ChpG^C^ exhibits diminished activity falling below the threshold necessary for recognition in eggplant. This hypothesis is supported by computational folding prediction analyses that suggest that V169G substitution alters folding stability, potentially resulting in diminished activity due to improper folding.

To assess the effect of the V169G substitution on protein function, we tested serine hydrolase activity *in vitro* and *in vivo* using several independent methodologies but were unable to detect activity in either the recognized ChpG^A^ variant or the ChpG^C^ variant. These methodologies include monitoring the cleavage of the Ala-Ala-Pro-Phe peptide, which serves as a standard substrate for chymotrypsin-type serine proteases; hydrolysis of azocasein, which serves as a less specific protease substrate; and a fluorescent serine hydrolase active site probe, which can detect the activity of various serine hydrolase types, including peptidases, amidases, and esterases [73–75]. The S231A substitution within the serine hydrolase catalytic triad in ChpG homologs of Cm and *C*. *capsici* resulted in a loss of activity regarding HR elicitation and virulence [39,46]. This suggests that ChpG is an active serine hydrolase and is unlikely to function as a pseudoprotease [76]. With that in mind, several scenarios may explain the inability to detect serine hydrolase activity. Assuming that, in accordance to protein fold prediction, ChpG is an S1 type serine protease, it is possible that it harbors a very narrow or unique substrate specificity or is found in an inactive form and requires exogenous activation. Unique substrate specificity in secreted serine proteases in regard to host interactions has been previously reported in several serine protease-like proteins (Spl) of *Staphylococcus aureus* that target unique cell surface proteins and complement components to promote aggression [68]. This specificity has been hypothesized to result from disparities from the canonical arrangement of a typical serine hydrolase active site, causing these proteases to target exclusive substrates [77–80]. Another possibility is that ChpG is secreted in an inactive form and requires conditional or exogenous activation that is not met under the experimental conditions in which the assays were performed. Many proteases are produced in inactive zymogen forms and become active through post-translational modification by other enzymes or through self-modification during exposure to favorable conditions, mainly by cleavage [81]. Cleavage-based activation has been reported in alpha-lytic proteases, in which ChpG demonstrates the closest predicted structural homology [82], suggesting that ChpG may be activated in a similar manner. Alternatively, ChpG may be conditionally activated only in the presence of an allosteric interactor found in the plant apoplast environment. Such allosteric interaction-based activation by proteinaceous and non-proteinaceous cofactors has been reported in numerous serine proteases and is suggested to play a significant regulatory role in tuning protease activity [83–85]. Lastly, despite the predicted protein fold, it is possible that ChpG and other Chp/Pat-1 proteins do not harbor peptidase activity and have evolved other biochemical functions mediated by the catalytic triad active site, such as acyltransferase activity [86,87], possibly modulating host signaling, potency of antimicrobial compounds, or the cell wall structures through modification of host targets instead of degradation. Future studies regarding the structure, activation mode, stability, and substrate specificity of ChpG will illuminate its enzymatic function and the evasion mechanism of the ChpG^C^ variant.

Adaptive mutations and loss in avirulence elicitors have long been hypothesized as the Achilles’ heel of gene-for-gene-based resistance [88], and host range expansion associated with such adaptations was documented on numerous occasions in plant pathogenic bacteria [11,89,90]. A recent example can be found in the host range shift of *Xanthomonas euvesicatoria* pv. *perforans*, which was previously considered to be restricted to tomato, but has now extended its host range to pepper due to loss or mutation in the effector *avrBsT* [13,91]. Notably, C47 and C48 were originally isolated from tomato. However, we expect host range expansion through minor modification in *chpG* can still harbor potential a beneficial advantage since tomato and eggplant are cultivated in the same geographic regions in Israel and some growers routinely graft eggplants on tomato rootstocks [92,93]. In addition, the host range of these isolates may expand to widespread wild eggplant relatives that potentially recognize ChpG and function as a reservoir for the pathogen, such as Buffalobur (*Solanum rostratum*) and Silverleaf (*Solanum elaeagnifolium*) nightshades [94].

A secondary key finding identified by our analyses was the loss of the *chp/tomA* PAI in Cm non-pathogenic isolates. Non-pathogenic *Clavibacter* clones have been previously isolated from tomato plants in multiple studies. Phylogenetic analyses of these isolates clustered them as either new *Clavibacter* species or as phylogenetically distant Cm strains belonging to a different phyletic lineage than that of pathogenic Cm strains [95–97]. In contrast to previous findings, phylogenetic analysis of our Cm library clustered all five tomato-non-pathogenic isolates within the Cm lineage. Comparative genomic analysis identified that the *chp/tomA* PAI, which is considered as the major pathogenicity-associated feature of Cm, was absent in all five isolates. We initially hypothesized that the five non-pathogenic isolates were progenitor Cm strains that never acquired the *chp/tomA* PAI. However, phylogenetic analysis did not support this hypothesis and showed that all five tomato-non-pathogenic isolates clustered with the pathogenic Cm isolates and can be separated into at least three independent phyletic lineages. This suggests that the *chp/tomA* PAI was more likely lost in pathogenic Cm, transforming them into non-pathogenic strains, and that this event happened on at least three separate occasions. The *chp/tomA* PAI is flanked by two almost identical direct repeats of ~1.9 kb [30,52], which are found in a single copy in the tomato-non-pathogenic isolates, suggesting that the loss of *chp/tomA* PAI occurred through homologous recombination between these repeats. Looping out of the *chp/tomA* PAI was reported at least twice in the model strain NCPPB382, which resulted in the non-pathogenic strains CMM30-10 and Cmm27 [15,30]. In both cases, the loss of the *chp/tomA* PAI occurred unintentionally after exposing the bacteria to high voltage during transformation, supporting that such event is feasible and could occur in nature. Considering that the loss of the *chp/tomA* PAI was fixated on multiple occasions suggests that it might be beneficial to Cm in certain conditions. Reduced or abolished virulence, due to the loss of large genomic islands associated with pathogenicity, has been previously reported in clinical and field isolates of animal and plant pathogenic bacteria such as *Helicobacter pylori*, uropathogenic *Escherichia coli*, *Ralstonia solanacearum*, and *Xanthomonas arboricola* [98–100]. Despite its crucial role in growth and pathogenesis in the host, we can speculate on several scenarios in which the loss of the *chp/tomA* PAI is beneficial to Cm. The tomato-non-pathogenic isolates in our library originated from tomato plants and can sustain a population of 10^6^−10^7^ CFU/g with very little consequences to the host plant while pathogenic isolates severely damaged the host and, in some occasions, killed the host within weeks after inoculation. Maintaining lower endophytic populations in the host for longer periods might be beneficial to Cm in certain circumstances [101]. Alternatively, the presence of the *chp/tomA* PAI might restrict Cm from colonizing alternative hosts through recognition of putative secreted hydrolases that are specifically recognized in non-host plants such as ChpG or Pat-1 [36,38,46]. Therefore, losing the *chp/tomA* PAI might expand the number of hosts Cm can occupy as an endophyte. Another possibility is that isolates that lost the *chp/tomA* PAI originated from a cheater sub-population that utilized the *chp/tomA*-encoded putative secreted hydrolases of the tomato pathogenic isolates as public goods and eventually took over the population [102]. Further studies regarding the potential benefits of losing the *chp/tomA* PAI, such as competition assays in host and non-host plants will shed insights into the evolutionary mechanisms that maintain it.

Our study provided novel insights into the phenotypic complexity within the population of bacterial plant pathogens and utilized comparative genomic approaches to link phenotypic variants to distinct genetic features. In addition, we determined that the host range of specialized plant pathogen such as Cm is a variable feature that differs between clones within the population and in our case, is determined by a minor genetic alteration.

## Materials and methods

### Bacterial strains and plant material

*Clavibacter michiganensis* (Cm) isolates used in this study are listed in S1 Table. *E*. *coli* strains used in this study are DH5α (Invitrogen) and Rosetta (MERCK) which were utilized for cloning procedures and protein purification, respectively. *Agrobacterium tumefaciens* strain GV2260 [103] was used for plant transient expression assays.

Cm, *A*. *tumefaciens* and *E*. *coli* were grown in Luria Bertani (LB) broth at 28°C (for Cm, and *A*. *tumefaciens*) or 37°C (for *E*. *coli*). When required, media were supplemented with 10 μg/ml nalidixic acid, 10 μg/ml chloramphenicol, 75 μg/ml neomycin, 50 μg/ml kanamycin or 100 μg/ml ampicillin. Plant cultivars used in this study are tomato (*Solanum lycopersicum*) var. Moneymaker, eggplant (*Solanum melongena*) var. Black Queen, *Mirabilis jalapa*, and *Nicotiana benthamiana*. Plants were grown in a 25°C temperature-controlled glasshouse under natural light conditions.

### Assembly of the Cm isolate library

The Cm isolate library consists of 37 isolates which were selected from a collection of more than 250 clones originating from tomato plants displaying bacterial canker symptoms in different regions of Israel from 1994 to 2023. Details regarding the collection area, year of isolation, host type, and any additional information are listed in S1 Table. Clones that were isolated from 1994 to 2011 were classified into groups according to Pulsed-field gel electrophoresis (PFGE) fingerprinting profile by Dr. Shulamit Manulis-Sasson [21,48](S1 Table). To further diversify our isolate library, three additional clones (C20, C30, and C31) from a reference Cm strains collection were added as well. These clones were chosen because they had a unique PFGE profile that was different from the other Israeli isolates (S1 Table).

### Genome sequencing

Total DNA was extracted from 10 ml bacterial overnight grown cultures using Wizard Genomic DNA Purification Kit (Promega) according to manufacturer’s instructions. Genomic DNA of the Cm isolates was sequenced using an Illumina Nextseq2000 platform with 150bp paired-end reads at the Applied Microbiology Services Laboratory (The Ohio State University). Samples were cleaned using Trimmomatic [104] with default parameters and assembled using Unicycler 0.5.0 and SPAdes v. 3.15.5 [105,106]. Contigs smaller than 200bp were removed, and genome completeness was assessed using BUSCO v5 using micrococcales_odb10 lineage [107].

### Determining *chpG* allelic variants

To determine the presence and allelic variants of *chpG* in our isolates library we conducted a manual Standard Nucleotide BLAST alignment of CMM_0059 against the contigs of all isolates. The aligned DNA sequences of *chpG* ORFs of all isolates and their predicted amino acid sequences were than compared to each other using multiple sequence alignment tool in Clustal Omega (https://www.ebi.ac.uk/Tools/msa/clustalo/). Next, the presence/absence of *chpG* and its allelic variants were confirmed by PCR amplification followed by Sanger sequencing for isolates C3, C4, C5, C6, C18, C21, C29, C30, C31, C45, C46, C47, C48, C61, and C68. To that aim, 1399 bp fragments flanking between the 271 bp upstream to the *chpG* ORF and 294 bp downstream to the *chpG* ORF were amplified with Q5 high fidelity DNA polymerase (NEB) using gene specific primers (S3 Table). Each fragment was sequenced by Sanger at Hylabs laboratories using both forward and reverse primer, manually assembled and compared to the *chpG* genomic regions in the corresponding genomes. Specific descriptions of the different *chpG* variants and affiliation with isolates are present in S5 and S6 Figs and Table 1.

### Plant inoculations, disease severity assessments, and quantification of stem bacterial populations

The virulence assays were carried out using a method similar to that described by Verma and Teper, 2022 [46], with some minor adjustments. Stem inoculations were conducted by a single punctures of the stem areas between the cotyledons of three-leaf stage eggplants or four-leaf tomatoes with Cm-contaminated toothpicks. Contaminated toothpicks were prepared as follows: Cm bacteria were scraped from fresh 2-day-old cultures grown on LB agar and diluted to 5 × 10^7^ CFU/ml in distilled water in 1.5-ml tubes. Wooden toothpicks were soaked in solutions for at least 10 min and a single toothpick was then used for a single inoculation. After inoculations, plants were kept at 25°C in a glasshouse under natural light conditions and wilt/leaf blotch symptoms were determined and scored at 14 dpi.

Wilting/leaf blotch symptoms were scored in each plant as the percentage of leaves demonstrating wilting and/or necrotic blotch symptoms according to the following scale: 0 = no wilt or leaf blotch, 1 = 1%–25%, 2 = 26%–50%, 3 = 51%–100%.

Bacterial populations were quantified in 1-mm stem areas taken 1 cm above the inoculation sites. Samples were weighed and supplemented with 1 ml of sterile distilled water. Samples were homogenized and bacterial numbers per gram of tissue were determined by plating 10 μl of 10-fold serial dilutions and counting the resulting colonies.

### Plasmid construction and bacterial transformation

Details regarding plasmid construction and primer sequences are available in S3 and S4 Tables. The pMA-RQ:Cmp plasmid, which was used for sub-cloning of *chpG* variants, was synthesized using GeneArt services (ThermoFisher) and contains a 411 bp fragment composed of the *pCMP1* promoter (50302–50044 bp region of CMP1 NCBI GenBank GQ241246) followed by a multiple cloning site and a triple HA tag. To construct *Clavibacter chpG* expression vectors, *chpG* ORFs were amplified from Cm^101^ (Var’ A), C5 (Var’ B1), C29 (Var’ B2), C48 (Var’ C), and C6 (Var’ D), and cloned into pMA-RQ:Cmp. For site directed mutagenesis, Val169 of ChpG^A^ and Gly169 of ChpG^C^ were substituted to glycine and valine, respectively, using the QuikChange II kit (Agilent Technologies). The *pCMP1*:*chpG*:3XHA units were cloned into the *E*. *coli*-*Clavibacter* shuttle vector pHN216 [51] and transformed into Cm^101^Ω*chpG*, Cm^101^ΔPAI and C48 as described in Verma and Teper, 2022 [46]. Protein accumulation was monitored in lysed overnight Cm cultures by western blot using HA Tag Monoclonal Antibody (2–2.2.14, ThermoFisher) as described by Sambrook and Russell, 2006 [108], and according to the manufacturer’s instructions.

### Expression and purification of MBP fusion proteins in *E*. *coli*

For construction of *MBP-chpG* fusions plasmids, 112–831 bp fragments containing *chpG* ORFs (CMM_0059) minus the signal peptide-coding region (predicted by SignalP-5.0 Server; http://www.cbs.dtu.dk/services/SignalP/) were amplified from genomic DNA of Cm^101^, genomic DNA of C48, and pHN216:*ChpG*^A^_S231A_-HA plasmid DNA, and cloned into pMAL-p5x (NEB). Plasmids were introduced into *E*. *coli* Rosetta cells. Details regarding plasmid construction and primer sequences are available in S3 and S4 Tables.

For protein purification, bacterial cultures were grown in an orbital shaker at 37°C until reaching OD6_00_ = 0.4–0.6, supplemented with 0.1 mM isopropyl β-d-1-thiogalactopyranoside, and incubated for 4 h at 37°C. Bacteria were pelleted and resuspended in ice-cold buffer solution (20 mM Tris-HCl, 200 mM NaCl, 1 mM EDTA, pH 7.4) and lysed using a SONIC-150W ultrasonic processor (MRC Labs). MBP-fused proteins were purified from supernatants using amylose resin (NEB) according to the manufacturer’s instructions. Purified proteins were quantified by Bradford protein assay kit (Bio-Rad) and validated by SDS-PAGE followed by staining with Coomassie brilliant blue. Protein accumulation in the purified fractions was confirmed with western blot using anti-MBP tag (8G1) mouse monoclonal antibody (Cell Signaling) according to the manufacturer’s instructions.

### *Agrobacterium*-mediated transient expression

Binary vectors were constructed for *Agrobacterium*-mediated transient expression of apoplastic *chpG* variants under the control of the *CaMV35S* promoter. First, we modified the pBTEX binary vector [109] by replacing the MCS with a eukaryote signal peptide followed by an alternative MCS and a triple HA tag to produce pBTEX sHA. The MCS + 3×HA of pMA-RQ:Cmp was amplified by PCR and cloned into the KpnI/SalI sites of pBTEX. Next, the 1–99 bp fragment of NtPR1a (accession num’ X06930), representing the 33 amino acid secretion signal sequence, was generated using overlap PCR and cloned into the KpnI/BamHI sites. Mature (112–831 bp fragments) variants of ChpG^A^, ChpG^C^ and ChpG^A^_S231A_ were amplified from genomic DNA of Cm^101^, C48 and the pHN216:ChpG^A^_S231A_-HA plasmid [46], and cloned into the BamHI/XbaI sites of pBTEX sHA. Plasmids were introduced into *Agrobacterium tumefaciens* GV2260 strain by electroporation.

For transient expression, *Agrobacterium* overnight cultures were pelleted, resuspended in induction medium (10 mM MgCl_2_, 10 mM MES pH 5.6, 200 mM acetosyringone), and incubated at 25°C with shaking for 4 h. Bacterial cultures were diluted to OD_600_ = 0.2 and infiltrated into leaves of eggplant and *Nicotiana benthamiana*. Protein accumulation was monitored in leaf tissues 72 h post infiltration. For protein extraction, 3 leaf disks of 1 cm diameter were frozen in liquid nitrogen, homogenized in extraction buffer (100 mM Tris pH 7.4, 1% Triton X-100)_,_ and centrifuged. Supernatants were separated by SDS-PAGE and protein expression was confirmed western blot using HA Tag Monoclonal Antibody (2–2.2.14, ThermoFisher) as described by Sambrook and Russell, 2006 [108] and according to the manufacturer’s instructions.

### Leaf infiltrations and ion leakage measurements

Purified proteins or bacterial suspensions were infiltrated into fully expanded upper rosette leaves of four- to six-leaf stage Black Queen eggplant and *Mirabilis jalapa* using a needleless syringe. For protein infiltration, purified MBP, MBP-ChpG^A,B^, MBP-ChpG^A,B^_S231A_ or MBP-ChpG^C^ proteins were diluted to a concentration of 0.01 μg/ml in 10 mM MgCl_2_ prior to infiltration. For infiltrations with bacterial suspensions, Cm bacteria were scraped from fresh 2-day-old cultures grown on LB agar and diluted to a concentration of 10^8^ (OD_600_ = 0.1) in 10 mM MgCl_2_ prior to infiltration.

For ion leakage measurements, 1.5-cm diameter leaf disks were sampled from the inoculation sites, transferred to flasks containing 10 ml of distilled water, and incubated on an orbital shaker (50 rpm) for 4 h at room temperature. Electrolyte leakage was quantified in water solutions using a conductivity meter (MRC Labs).

### Protein structure prediction and molecular dynamic simulations

The 3D-structure of ChpG^A^ and ChpG^A^_V169G_ was predicted using the Artificial Intelligence-based Alpha-Fold2 through the AlphaFold Colab notebook platform [53]. For the structural comparisons, we used only the predicted model with the highest confidence score computed by AlphFold for each protein. PyMOL (PyMOL Molecular Graphics System, Version 2.4, Schrödinger) was used to examine the structural models and generate images. Molecular dynamics simulations of ChpG^A^ and ChpG^A^_V169G_ was performed with GROMACS [110] using the online WEBGRO Macromolecular Simulations server (https://simlab.uams.edu/ProteinWithLigand/protein_with_ligand.html). We preformed two 50ns simulation for each of the structures with default settings.

### *In vitro* peptidase activity assays

*In vitro* peptidase activity assays were conducted using purified MBP-ChpG^A,B^, MBP-ChpG^C^, MBP-ChpG^A/B^_S231A_, MBP and proteinase K (positive control)(Sigma, CAS Number:39450-01-6).

Cleavage of the chymotrypsin colorimetric substrate N-Succinyl-Ala-Ala-Pro-Phe-p-nitroanilide (N-AAPF-PN)(Sigma, CAS Number:70967-97-4) was conducted as follows: 1 μg MBP fusion proteins or 0.1 μg proteinase K were incubated in 100 μl protease activity solution (1 mM N-AAPF-PN, 50 mM Tris HCl, pH 7.4) at 25°C for 1 h. Substrate cleavage was determined by measuring free p-nitroaniline at 410 nm.

Azocasein (Sigma, CAS No.: 102110-74-7) hydrolysis was conducted as follows: 1 μg MBP fusion proteins or 0.1 μg proteinase K were incubated in 1500 μl Azocasein hydrolysis solution (1% Azocasein, 50 mM Tris-HCl buffer, pH 8.0, 10 mM CaCl_2_) for 1 h. Next, 1000 μl solution mix was supplemented with 500 μl 110 mM Trichloroacetic Acid and centrifuged at 20,000 rpm for 20 min. Next, 500 μl supernatant was mixed with 500 μl 0.5 N NaOH. Azocasein hydrolysis was determined by measuring absorbance at 450 nm.

### Serine hydrolase activity assays using active site probe

Serine hydrolase activity assays were conducted using ActivX TAMRA-FP Serine Hydrolase Probe (ThermoFisher scientific) [73]. Assays were conducted with purified MBP-fused ChpG protein variants, total protein culture media or supernatants of Cm^101^ΔPAI expressing *chpG* variants, total *N*. *benthamiana* leaf protein extract transiently expressing *chpG* variants.

For Cm^101^ΔPAI-based culture protein extraction, bacteria were grown in LB broth for 18 hours at 28°C, followed by extraction of total protein from 1 ml and collection of the culture supernatant. Extraction of total protein was conducted by lysing the cultures using a SONIC-150W ultrasonic processor, followed by centrifugation, collection of the supernatant, and supplementation with 1:10 10X Tris-buffered saline (TBS), pH 7.5. For isolation of secreted proteins, cultures were centrifuged at 14,000 rpm for 10 minutes, supernatants were collected, filtered through a 0.22 μm nylon membrane, and supplemented with 1:10 10X TBS.

For isolation of *N*. *benthamiana* protein extracts, leaves were infiltrated with *Agrobacterium* carrying binary vectors for transient expression of *chpG* variants. Total protein was extracted from pooled samples of three 1.5 cm diameter leaf disks 72 hours post-infiltration by homogenizing the samples in 500 μl TBS, followed by centrifugation and collection of the supernatants.

The TAMRA-FP probe was diluted to 0.2 μM in 50 μl Cm cultures, Cm supernatants, leaf extracts, TBS with 1 μg MBP fusion proteins, or TBS with 0.1 μg proteinase K and incubated for one hour. Next, samples were separated on SDS-PAGE. Active site fluorescent binding was visualized on the gel using a Sapphire Biomolecular Imager (Azure Biosystems) with excitation at 530 nm and emission at 554 nm.

## Supporting information

S1 Fig *Clavibacter michiganensis* (Cm) isolates demonstrate differential virulence in tomato. Four-leaf stage “Moneymaker” tomato plants were inoculated with the indicated Cm isolates or water control (mock) by puncturing the stem area between the cotyledons with a wooden toothpick incubated in *Cm* solution (5 × 10^7^ CFU/ml). Representative plants were photographed 14 days post inoculations. Experiments were repeated at least twice using 3–5 plants for each of the tested Cm isolates.
(PDF)

S2 Fig *Clavibacter michiganensis* (Cm) isolates demonstrate differential virulence in eggplant. Three-leaf stage “Black Queen” eggplants were inoculated with the indicated *Clavibacter michiganensis* (Cm) isolates or water control (mock) by puncturing the stem area between the cotyledons with a wooden toothpick incubated in *Cm* solution (5 × 10^7^ CFU/ml). Representative plants were photographed 14 days post inoculations. Experiments were repeated at least twice using 3–5 plants for each of the tested Cm isolates.
(PDF)

S3 Fig Genome sequence alignment of tomato-pathogenic *Clavibacter michiganensis* (Cm) isolates. Whole genome alignments of the tomato pathogenic Cm isolates were done against CDS of the Cm strain NCPPB382 chromosome (NCBI GenBank: AM711867), the pCM1 plasmid (AM711865), and the pCM2 plasmid (AM711866), and visualized using BLAST atlas analysis in Gview server (https://server.gview.ca/) using default features. The *chp/tomA* island, *celA* (pCM1_0020) and *pat-1* (pCM2_0054) are respectively marked in the chromosome, pCM1, and pCM2 alignments. (**A**) Isolates: C3, C4, C5, C6, C8, C18, C20, C21, C22, C23, and C25. (**B**) Isolates: C26, C29, C30, C31, C32, C33, C34, C37, C38, and C39. (**C**) Isolates: C40, C41, C42, C43, C44, C45, C46, C47, C48, and C49. (**D**) Isolates: C50, C53, C54, C55, C56, C58, C59, C61, C68, and C70.
(PDF)

S4 Fig Global alignment of the genes surrounding the *chp/tomA* island. The colors indicate homologous gene groups. All coded regions have over 60% alignment sequence identity. Clinker v0.0.27 was used to create the figure, using protein translations predicted by Prokka v1.14.5. Isolates which were pathogenic on tomato but not pathogenic on eggplant are labeled in red, isolates which were pathogenic on tomato and eggplant are labeled in a purple, and isolates which were non-pathogenic on tomato and eggplant are labeled in blue.
(PDF)

S5 Fig DNA sequence alignment of *chpG* homologs. The five *Clavibacter michiganensis chpG* homologs were aligned by Clustal Omega multiple sequence alignment tool (https://www.ebi.ac.uk/Tools/msa/clustalo/) using default features. Polymorphic site are marked with green (common polymorphic site) or magenta (rare polymorphic site).
(DOCX)

S6 Fig Amino acid sequence alignment of ChpG homologs. The four Cm ChpG homologs were aligned by Clustal Omega multiple sequence alignment tool (https://www.ebi.ac.uk/Tools/msa/clustalo/) using default features. Amino acid polymorphic sites are marked with green (common polymorphic site) or magenta (rare polymorphic site).
(DOCX)

S7 Fig Protein accumulation of ChpG variants in Cm^101^Ω*chpG* and Cm^C48^. Overnight cultures of *Clavibacter michiganensis* clones carrying pHN216 with the indicated inserts expressed under the pCMP1 promoter were diluted to OD_600_ = 0.5, lysed with protein sample buffer and separated on SDS-PAGE and gels were either stained with Coomassie brilliant blue (**A**, **B**, **C**, **bottom panel**) or transferred to nitrocellulose membrane and immunoblotted with anti-HA antibody (**A**, **B**, **C**, **top panel**).
(PDF)

S8 Fig Introduction of *ChpG*^A^ and *ChpG*^C^ into Cm^C48^ or Cm^101^Ω*chpG* does not affect virulence on tomato. Four-leaf stage "Moneymaker" tomato plants were inoculated with the indicated *Clavibacter michiganensis* (Cm) clones or water control (mock) by puncturing the stem area between the cotyledons with a wooden toothpick incubated in *Cm* solution (5 × 10^7^ CFU/ml). (**A, B**) Representative plants were photographed 21 days post inoculations (dpi). (**C**) Wilting symptoms were quantified at 21 dpi according to the following scale: 0 = no wilting, 1 = 1–25%, 2 = 25–50%, 3 = 50–100%. Graph depicts the symptom distribution in at least nine plants pooled from two independent experiments. (**D**) Stem bacterial populations 1 cm above the inoculation site at 21 dpi. Lower and upper quartiles are marked at the margins of the boxes. Central lines and “o” represent medians and data points of at least nine biological repeats collected from two independent experiments.
(PDF)

S9 Fig Comparative analyses of the predicted 3D structures of ChpG^A^ and ChpGAV169G. (**A**) The 3D-structures of ChpG^A^ (marked in aqua) and ChpG^A^_V169G_ (marked in green) were predicted using Alpha-Fold2 through the AlphaFold Colab notebook platform. Structure alignment was produced using PyMOL. Enlarged rectangle represent structure shift in the 161–170 aa region. The 169 position is marked in magenta. (**B**) Root Mean Square Fluctuation (RMSF), representing the level fluctuation of individual residues from their average positions over a simulation trajectory of ChpG^A^ (aqua) and ChpG^A^_V169G_ (green) using the online WEBGRO Macromolecular Simulations server (https://simlab.uams.edu/ProteinWithLigand/protein_with_ligand.html). Simulation was repeated twice with similar results.
(PDF)

S10 Fig Leaf infiltrations of Cm^101^ΔPAI and *Agrobacterium* carrying different *chpG* variants. (**A**) Five to six leaf-stage ’Black Queen’ eggplant leaves were infiltrated (10^8^ CFU/ml) with Cm^101^ΔPAI expressing the indicated *chpG* variants or an empty vector control (EV). A representative photograph was taken 48 hours post-infiltration. (**B**) ’Black Queen’ eggplant (**left panel**) and *Nicotiana benthamiana* (**right panel**) leaves were infiltrated with *Agrobacterium* strains carrying vectors aimed at transient expression of the indicated mature *chpG* variants fused to the 33 aa secretion signal of NtPR1 at the N-terminus under the expression of the *CaMV35S* promoter. The 33 aa secretion signal of NtPR1 alone was used as an empty vector control (EV). Representative photographs were taken 72 hours post-infiltration. Photographs represent at least 15 repeats with similar results taken from at least two independent experiments.
(PDF)

S1 Table Cm clones used in this study. (DOCX)

S2 Table Sequencing statistics of Cm isolates sequenced during this study. (DOCX)

S3 Table Primers used during this study. (DOCX)

S4 Table Plasmids used in this study. (DOCX)
