# Supplementary material for: Allelic variations in the chpG effector gene within Clavibacter michiganensis populations determine pathogen host range
Source: PLoS Pathog. 2024 Jul 19;20(7):e1012380. doi: 10.1371/journal.ppat.1012380 (PMC11290698; doi:10.1371/journal.ppat.1012380)
Supplement: S1 Fig — Four-leaf stage “Moneymaker” tomato plants were inoculated with the indicated Cm isolates or water control (mock) by puncturing the stem area between the cotyledons with a wooden toothpick incubated in Cm solution (5 × 10^7^ CFU/ml). Representative plants were photographed 14 days post inoculations. Experiments were repeated at least twice using 3–5 plants for each of the tested Cm isolates. (PDF) [file ppat.1012380.s001.pdf]

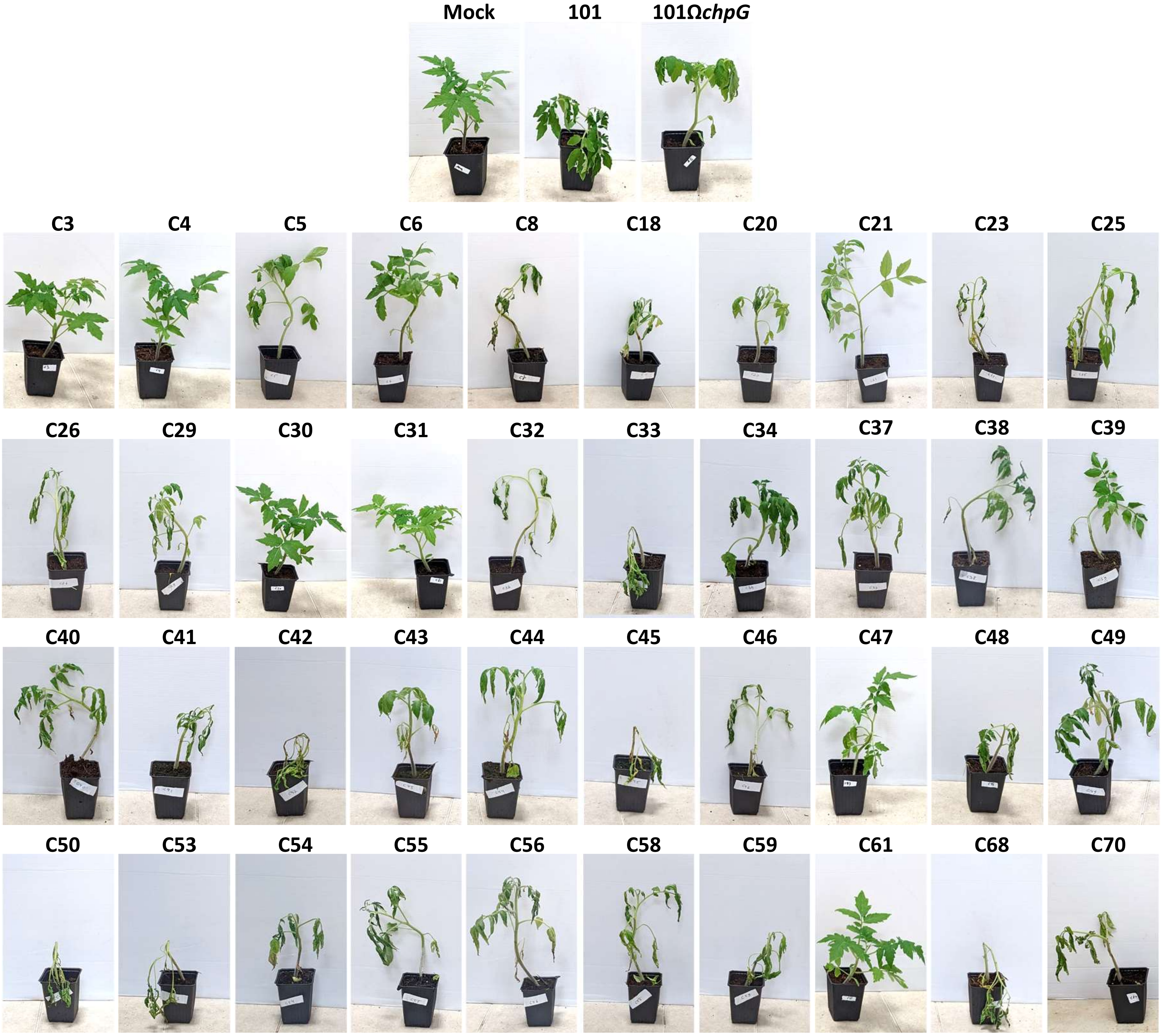

S1 Figure. *Clavibacter michiganensis* (Cm) isolates demonstrate differential virulence in tomato. Four-leaf stage "Moneymaker" tomato plants were inoculated with the indicated Cm isolates or water control (mock) by puncturing the stem area between the cotyledons with a wooden toothpick incubated in *Cm* solution (5×10^7 CFU/ml). Representative plants were photographed 14 days post inoculations. Experiments were repeated at least twice using 3-5 plants for each of the tested Cm isolates.
