# Supplementary material for: Allelic variations in the chpG effector gene within Clavibacter michiganensis populations determine pathogen host range
Source: PLoS Pathog. 2024 Jul 19;20(7):e1012380. doi: 10.1371/journal.ppat.1012380 (PMC11290698; doi:10.1371/journal.ppat.1012380)
Supplement: S3 Fig — Whole genome alignments of the tomato pathogenic Cm isolates were done against CDS of the Cm strain NCPPB382 chromosome (NCBI GenBank: AM711867), the pCM1 plasmid (AM711865), and the pCM2 plasmid (AM711866), and visualized using BLAST atlas analysis in Gview server (https://server.gview.ca/) using default features. The chp/tomA island, celA (pCM1_0020) and pat-1 (pCM2_0054) are respectively marked in the chromosome, pCM1, and pCM2 alignments. (A) Isolates: C3, C4, C5, C6, C8, C18, C20, C21, C22, C23, and C25. (B) Isolates: C26, C29, C30, C31, C32, C33, C34, C37, C38, and C39. (C) Isolates: C40, C41, C42, C43, C44, C45, C46, C47, C48, and C49. (D) Isolates: C50, C53, C54, C55, C56, C58, C59, C61, C68, and C70. (PDF) [file ppat.1012380.s003.pdf]

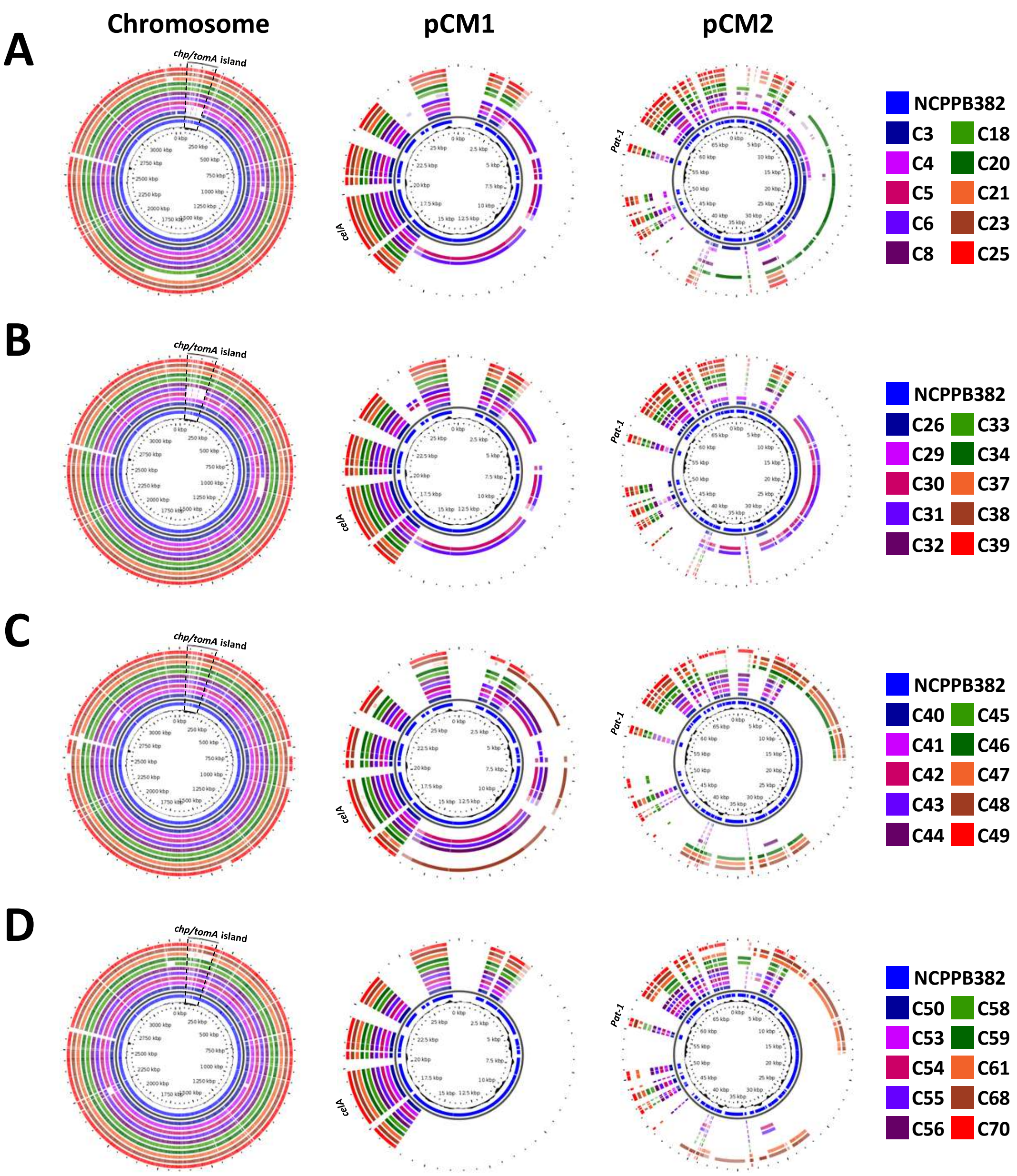

S3 Figure. Genome sequence alignment of tomato-pathogenic *Clavibacter michiganensis* (Cm) isolates. Whole genome alignment of the tomato pathogenic Cm isolates was done against CDS of Cm strain NCPPB382 chromosome (NCBI GenBank: AM711867), pCM1 plasmid (AM711865), and pCM2 plasmid (AM711866), and was visualized using BLAST atlas analysis in Gview server (https://server.gview.ca/) using default features. The *chp/tomA* island, *celA* (pCM1_0020) and *pat-1* (pCM2_0054) are respectively marked in the chromosome, pCM1, and pCM2 alignments. (**A**) Isolates: C3, C4, C5, C6, C8, C18, C20, C21, C22, C23 and C25. (**B**) Isolates: C26, C29, C30, C31, C32, C33, C34, C37, C38 and C39. (**C**) Isolates: C40, C41, C42, C43, C44, C45, C46, C47, C48 and C49. (**D**) Isolates: C50, C53, C54, C55, C56, C58, C59, C61, C68, and C70.
