# Supplementary material for: Allelic variations in the chpG effector gene within Clavibacter michiganensis populations determine pathogen host range
Source: PLoS Pathog. 2024 Jul 19;20(7):e1012380. doi: 10.1371/journal.ppat.1012380 (PMC11290698; doi:10.1371/journal.ppat.1012380)
Supplement: S4 Fig — The colors indicate homologous gene groups. All coded regions have over 60% alignment sequence identity. Clinker v0.0.27 was used to create the figure, using protein translations predicted by Prokka v1.14.5. Isolates which were pathogenic on tomato but not pathogenic on eggplant are labeled in red, isolates which were pathogenic on tomato and eggplant are labeled in a purple, and isolates which were non-pathogenic on tomato and eggplant are labeled in blue. (PDF) [file ppat.1012380.s004.pdf]

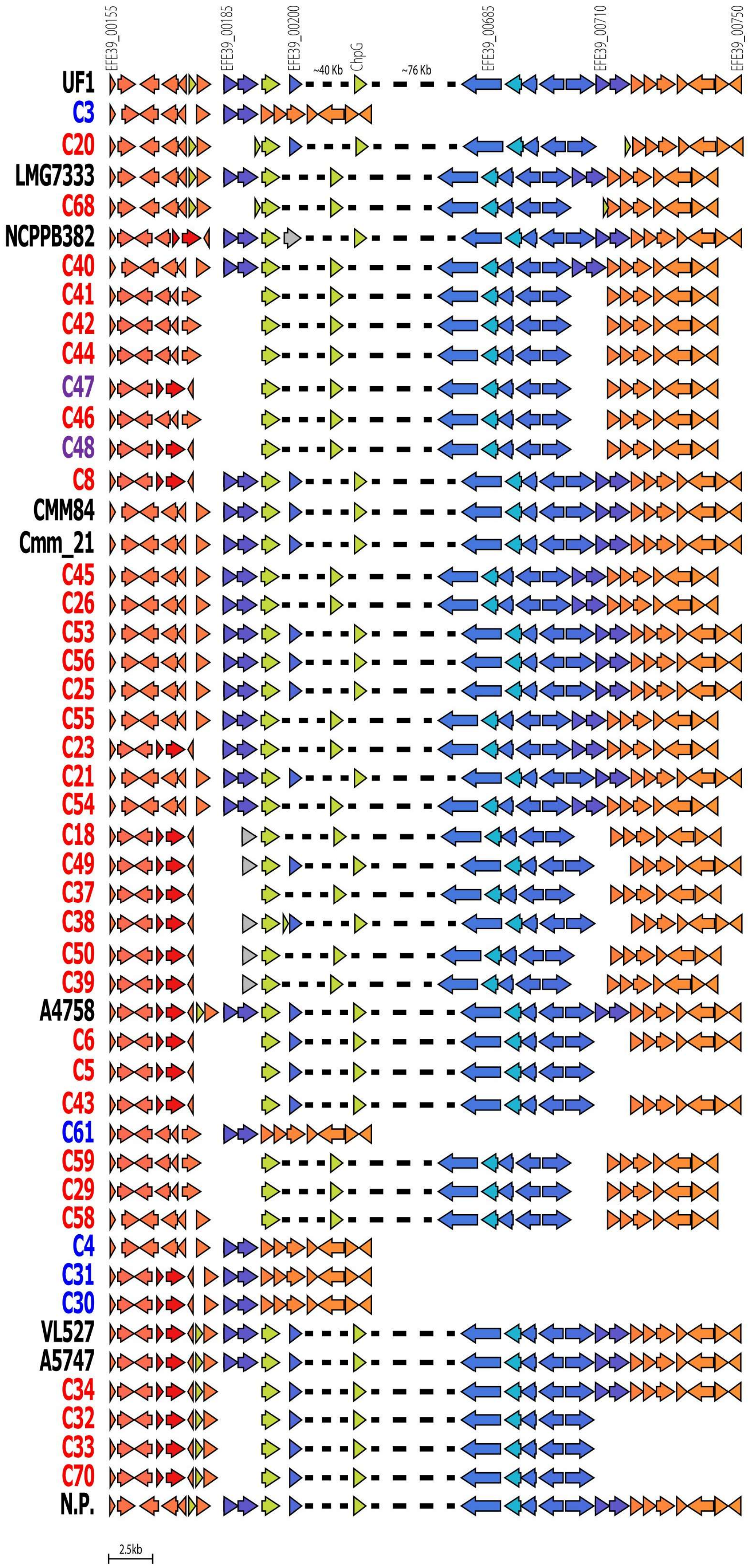

S4 Figure. Global alignment of the genes surrounding the *chp/tomA* island. The colors indicate homologous gene groups. All coded regions have over 60% alignment sequence identity. Clinker v0.0.27 was used to create the figure, using protein translations predicted by Prokka v1.14.5. Isolates which were pathogenic on tomato but not pathogenic on eggplant are labeled in red, isolates which were pathogenic on tomato and eggplant are labeled in a purple, and isolates which were non-pathogenic on tomato and eggplant are labeled in blue.
