# Supplementary material for: Allelic variations in the chpG effector gene within Clavibacter michiganensis populations determine pathogen host range
Source: PLoS Pathog. 2024 Jul 19;20(7):e1012380. doi: 10.1371/journal.ppat.1012380 (PMC11290698; doi:10.1371/journal.ppat.1012380)
Supplement: S5 Fig — The five Clavibacter michiganensis chpG homologs were aligned by Clustal Omega multiple sequence alignment tool (https://www.ebi.ac.uk/Tools/msa/clustalo/) using default features. Polymorphic site are marked with green (common polymorphic site) or magenta (rare polymorphic site). (DOCX) [file ppat.1012380.s005.docx]

chpG^A1^ TTGCCTGCTCGCCATCACACCATCCAGCGCAAGCGCTCAATAGGGGCTGCTCTTCTCGCT 60

chpG^B1^ TTGCCTGCTCGCCATCACACCATCCAGCGCAAGCGCTCAATAGGGGCTGCTCTTCTCGCT 60

chpG^B2^ TTGCCTGCTCGCCATCACACCATCCAGCGCAAGCGCTCAATAGGGGCTGCTCTTCTCGCT 60

chpG^C1^ TTGCCTGCTCGCCATCACACCATCCAGCGCAAGCGCTCAATAGGGGCTGCTCTTCTCGCT 60

chpG^D1^ TTGCCTGCTCGCCATCACACCATCCAGCGCAAGCGCTCAATAGGGGCTGCTCTTCTCGCT 60

************************************************************

chpG^A1^ CTGCCGGCGACCCTTGTCTTGACCTGCATGGCGGGAACACCCGCCTACGCGAACGGACTC 120

chpG^B1^ CTGCTGGCGACCCTTGTCTTGACCTGCATGGCGGGAACACCCGCCTACGCGAACGGACTC 120

chpG^B2^ CTGCTGGCGACCCTTGTTTTGACCTGCATGGCGGGAACACCCGCCTACGCGAACGGACTC 120

chpG^C1^ CTGCTGGCGACCCTTGTCTTGACCTGCATGGCGGGAACACCCGCCTACGCGAACGGACTC 120

chpG^D1^ CTGCTGGCGACCCTTGTCTTGACCTGCATGGCGGGAACACCCGCCTACGCGAACGGACTC 120

**** ************ ******************************************

chpG^A1^ AGCAACCCGGACCGCGGAAACTTCCCCATCATCGCCGGTTCCGAAGTCGGCGTTCCGAAT 180

chpG^B1^ AGCAACCCGGACCGCGGAAACTTCCCCATCATCGCCGGTTCCGAAGTCGGCGTTCCGAAT 180

chpG^B2^ AGCAACCCGGACCGCGGAAACTTCCCCATCATCGCCGGTTCCGAAGTCGGCGTTCCGAAT 180

chpG^C1^ AGCAACCCGGACCGCGGAAACTTCCCCATCATCGCCGGTTCCGAAGTCGGCGTTCCGAAT 180

chpG^D1^ AGCAACCCGGACCGCGGAAACTTCCCCATCATCGCCGGTTCCGAAGTCGGCGTTCCGAAT 180

************************************************************

chpG^A1^ GGCTACTGCAGCGTCGGAGCCGTGCTCGTTCCCAGCAGCATCTTCCAGCGGATCACCCCA 240

chpG^B1^ GGCTACTGCAGCGTCGGAGCCGTGCTCGTTCCCAGCAGCATCTTCCAGCGGATCACCCCA 240

chpG^B2^ GGCTACTGCAGCGTCGGAGCCGTGCTCGTTCCCAGCAGCATCTTCCAGCGGATCACCCCA 240

chpG^C1^ GGCTACTGCAGCGTCGGAGCCGTGCTCGTTCCCAGCAGCATCTTCCAGCGGATCACCCCA 240

chpG^D1^ GGCTACTGCAGCGTCGGAGCCGTGCTCGTTCCCAGCAGCATCTTCCAGCGGATCACCCCA 240

************************************************************

chpG^A1^ TATCAGCGCGCTGTTCGCTACCTCGTCCTCGCCAAGCACTGCGCTCCGCTCAACTCGCCC 300

chpG^B1^ TATCAGCGCGCTGTTCGCTACCTCGTCCTCGCCAAGCACTGCGCTCCGCTCAACTCGCCC 300

chpG^B2^ TATCAGCGCGCTGTTCGCTACCTCGTCCTCGCCAAGCACTGCGCTCCGCTCAACTCGCCC 300

chpG^C1^ TATCAGCGCGCTGTTCGCTACCTCGTCCTCGCCAAGCACTGCGCTCCGCTCAACTCGCCC 300

chpG^D1^ TATCAGCGCGCTGTTCGCTACCTCGTCCTCGCCAAGCACTGCGCTCCGCTCAACTCGCCC 300

************************************************************

chpG^A1^ ATCTACTTCGCGCAGCAGGACATCGGAGACGTCGTCTGGCAGTCAGCAGCATCTGACATC 360

chpG^B1^ ATCTACTTCGCGCAGCAGGACATCGGAGACGTCGTCTGGCAGTCAGCAGCATCTGACATC 360

chpG^B2^ ATCTACTTCGCGCAGCAGGACATCGGAGACGTCGTCTGGCAGTCAGCAGCATCTGACATC 360

chpG^C1^ ATCTACTTCGCGCAGCAGGACATCGGAGACGTCGTCTGGCAGTCAGCAGCATCTGACATC 360

chpG^D1^ ATCTACTTCGCGCAGCAGGACATCGGAGACGTCGTCTGGCAGTCAGCAGCATCTGACATC 360

************************************************************

chpG^A1^ GAGCTGGTCCGCGTATCGCCCTCGCGCGACAACATGACCCTGCACTGCGCCGGCCACTCG 420

chpG^B1^ GAGCTGGTCCGCGTATCGCCCTCGCGCGACAACATGACCCTGCACTGCGCCGGCCACTCC 420

chpG^B2^ GAGCTGGTCCGCGTATCGCCCTCGCGCGACAACATGACCCTGCACTGCGCCGGCCACTCC 420

chpG^C1^ GAGCTGGTCCGCGTATCGCCCTCGCGCGACAACATGACCCTGCACTGCGCCGGCCACTCC 420

chpG^D1^ GAGCTGGTCCGCGTATCGCCCTCGCGCGACAACATGACCCTGCACTGCGCCGGTCACTCC 420

***************************************************** *****

chpG^A1^ AC---CCCCGCAACATGCAGCCCGATCCAGACCTTCACCCCTCGAGCCAACGGCCAAGTC 477

chpG^B1^ AC---CCCCGCAACATGCAGCCCGATCCAGACCTTCACCCCTCGAGCCAACGGCCAAGTC 477

chpG^B2^ AC---CCCCGCAACATGCAGCCCGATCCAGACCTTCACCCCTCGAGCCAACGGCCAAGTC 477

chpG^C1^ AC---CCCCGCAACATGCAGCCCGATCCAGACCTTCACCCCTCGAGCCAACGGCCAAGTC 477

chpG^D1^ ACGAACCCTGCAACATGCAGCCTGATCCAGACCTTCACCCCTCGAGCCAACAGCCAAGTC 480

** *** ************* **************************** ********

chpG^A1^ TTCATGACCGCACCGCCCTCACCGATCGTCGGGCGGCGAGCGATCGCAGGGACGGGTATT 537

chpG^B1^ TTCATGACCGCACCGCCCTCACCGATCGTCGGGCGGCGAGCGATCGCAGGGACGGGTATT 537

chpG^B2^ TTCATGACCGCACCGCCCTCACCGATCGTCGGGCGGCGAGCGATCGCAGGGACGGGTATT 537

chpG^C1^ TTCATGACCGCACCGCCCTCACCGATCGGCGGGCGGCGAGCGATCGCAGGGACGGGTATT 537

chpG^D1^ TTCATGACCGCGCCGCCCTCACCGATCGTCGGGCGGCGAGCGATCGCAGGGACGGGTATT 540

*********** **************** *******************************

chpG^A1^ CCGTCGGCCACGGGCACGTTCTGCACGAGCGGGCACGTCACCGGCGTCATCTGCGACTTC 600

chpG^B1^ CCGTCGGCCACGGGCACGTTCTGCACGAGCGGGCACGTCACCGGCGTCATCTGCGACTTC 597

chpG^B2^ CCGTCGGCCACGGGCACGTTCTGCACGAGCGGGCACGTCACCGGCGTCATCTGCGACTTC 597

chpG^C1^ CCGTCGGCCACGGGCACGTTCTGCACGAGCGGGCACGTCACCGGCGTCATCTGCGACTTC 597

chpG^D1^ CCGTCGGCCACGGGCACGTTCTGCACGAGCGGGCACGTCACCGGCGTCATCTGCGACTTC 600

************************************************************

chpG^A1^ CAGCCCACGAGCCTGCCTGTCGGGGTCCTCAGGGCGTATGAGCACCTTGCGGCTGGACAG 657

chpG^B1^ CAGCCCACGAGCCTGCCTGTCGGGGTCCTCAGGGCGTATGAGCACCTTGCGGCTGGACAG 657

chpG^B2^ CAGCCCACGAGCCTGCCTGTCGGGGTCCTCAGGGCGTATGAGCACCTTGCGGCTGGACAG 657

chpG^C1^ CAGCCCACGAGCCTGCCTGTCGGGGTCCTCAGGGCGTATGAGCACCTTGCGGCTGGACAG 657

chpG^D1^ CAGCCCACGAGCCTGCCTGTCGGGGTCCTCAGGGCGTATGAGCACCTTGCGGCTGGACAG 660

************************************************************

chpG^A1^ TCAGCCGCCGTGGGAGCGCTGCGGCCCGGCGACTCCGGCGGCCCCGTCGTCAGCAAGGAC 717

chpG^B1^ TCAGCCGCCGTGGGAGCGCTGCGGCCCGGCGACTCCGGCGGCCCCGTCGTCAGCAAGGAC 717

chpG^B2^ TCAGCCGCCGTGGGAGCGCTGCGGCCCGGCGACTCCGGCGGCCCCGTCGTCAGCAAGGAC 717

chpG^C1^ TCAGCCGCCGTGGGAGCGCTGCGGCCCGGCGACTCCGGCGGCCCCGTCGTCAGCAAGGAC 717

chpG^D1^ TCAGCCGCCGTGGGAGCGCTGCGGCCCGGCGACTCCGGCGGCCCCGTCGTCAGCAAGGAC 720

************************************************************

chpG^A1^ AGGCGGCTGCTTGGCATCATCTCCGGCGACGTGCCGAACACTCACTTCCTTGTCTACACC 777

chpG^B1^ AGGCGGCTGCTTGGCATCATCTCCGGCGACGTGCCGAACACTCACTTCCTTGTCTACACC 777

chpG^B2^ AGGCGGCTGCTTGGCATCATCTCCGGCGACGTGCCGAACACTCACTTCCTTGTCTACACC 777

chpG^C1^ AGGCGGCTGCTTGGCATCATCTCCGGCGACGTGCCGAACACTCACTTCCTTGTCTACACC 777

chpG^D1^ AGGCGGCTGCTTGGCATCATCTCCGGCGACGTGCCGAACACTCACTTCCTTGTCTACACC 780

************************************************************

chpG^A1^ CCGATGGCGCAGGTCCTCCACGAACTGTCGAGCTACAAGCTCGCACCCGCCAACTGA 834

chpG^B1^ CCGATGGCGCAGGTCCTCCACGAACTGTCGAGCTACAAGCTCGCACCCGCCAACTGA 834

chpG^B2^ CCGATGGCGCAGGTCCTCCACGAACTGTCGAGCTACAAGCTCGCACCCGCCAACTGA 834

chpG^C1^ CCGATGGCGCAGGTCCTCCACGAACTGTCGAGCTACAAGCTCGCACCCGCCAACTGA 834

chpG^D1^ CCGATGGCGCAGGTCCTCCACGAACTGTCGAGCTACAAGCTCGCACCCGCCAACTGA 837

*********************************************************

**S5 Figure. DNA sequence alignment of *chpG* homologs.** The five *Clavibacter michiganensis* *chpG* homologs were aligned by Clustal Omega multiple sequence alignment tool (<https://www.ebi.ac.uk/Tools/msa/clustalo/>) using default features. Polymorphic site are marked with green (common polymorphic site) or magenta (rare polymorphic site).
