# Supplementary material for: Allelic variations in the chpG effector gene within Clavibacter michiganensis populations determine pathogen host range
Source: PLoS Pathog. 2024 Jul 19;20(7):e1012380. doi: 10.1371/journal.ppat.1012380 (PMC11290698; doi:10.1371/journal.ppat.1012380)
Supplement: S6 Fig — The four Cm ChpG homologs were aligned by Clustal Omega multiple sequence alignment tool (https://www.ebi.ac.uk/Tools/msa/clustalo/) using default features. Amino acid polymorphic sites are marked with green (common polymorphic site) or magenta (rare polymorphic site). (DOCX) [file ppat.1012380.s006.docx]

ChpG^A^ MPARHHTIQRKRSIGAALLALPATLVLTCMAGTPAYANGLSNPDRGNFPIIAGSEVGVPN 60

ChpG^B^ MPARHHTIQRKRSIGAALLALLATLVLTCMAGTPAYANGLSNPDRGNFPIIAGSEVGVPN 60

ChpG^C^ MPARHHTIQRKRSIGAALLALLATLVLTCMAGTPAYANGLSNPDRGNFPIIAGSEVGVPN 60

ChpG^D^ MPARHHTIQRKRSIGAALLALLATLVLTCMAGTPAYANGLSNPDRGNFPIIAGSEVGVPN 60

********************* **************************************

ChpG^A^ GYCSVGAVLVPSSIFQRITPYQRAVRYLVLAKHCAPLNSPIYFAQQDIGDVVWQSAASDI 120

ChpG^B^ GYCSVGAVLVPSSIFQRITPYQRAVRYLVLAKHCAPLNSPIYFAQQDIGDVVWQSAASDI 120

ChpG^C^ GYCSVGAVLVPSSIFQRITPYQRAVRYLVLAKHCAPLNSPIYFAQQDIGDVVWQSAASDI 120

ChpG^D^ GYCSVGAVLVPSSIFQRITPYQRAVRYLVLAKHCAPLNSPIYFAQQDIGDVVWQSAASDI 120

************************************************************

ChpG^A^ ELVRVSPSRDNMTLHCAGHST-PATCSPIQTFTPRANGQVFMTAPPSPIVGRRAIAGTGI 179

ChpG^B^ ELVRVSPSRDNMTLHCAGHST-PATCSPIQTFTPRANGQVFMTAPPSPIVGRRAIAGTGI 179

ChpG^C^ ELVRVSPSRDNMTLHCAGHST-PATCSPIQTFTPRANGQVFMTAPPSPIGGRRAIAGTGI 179

ChpG^D^ ELVRVSPSRDNMTLHCAGHSTNPATCSLIQTFTPRANSQVFMTAPPSPIVGRRAIAGTGI 180

********************* ***** *********.*********** **********

ChpG^A^ PSATGTFCTSGHVTGVICDFQPTSLPVGVLRAYEHLAAGQSAAVGALRPGDSGGPVVSKD 239

ChpG^B^ PSATGTFCTSGHVTGVICDFQPTSLPVGVLRAYEHLAAGQSAAVGALRPGDSGGPVVSKD 239

ChpG^C^ PSATGTFCTSGHVTGVICDFQPTSLPVGVLRAYEHLAAGQSAAVGALRPGDSGGPVVSKD 239

ChpG^D^ PSATGTFCTSGHVTGVICDFQPTSLPVGVLRAYEHLAAGQSAAVGALRPGDSGGPVVSKD 240

************************************************************

ChpG^A^ RRLLGIISGDVPNTHFLVYTPMAQVLHELSSYKLAPAN 277

ChpG^B^ RRLLGIISGDVPNTHFLVYTPMAQVLHELSSYKLAPAN 277

ChpG^C^ RRLLGIISGDVPNTHFLVYTPMAQVLHELSSYKLAPAN 277

ChpG^D^ RRLLGIISGDVPNTHFLVYTPMAQVLHELSSYKLAPAN 278

**************************************

**S6 Figure. Amino acid sequence alignment of ChpG homologs.** The four Cm ChpG homologs were aligned by Clustal Omega multiple sequence alignment tool (<https://www.ebi.ac.uk/Tools/msa/clustalo/>) using default features. Amino acid polymorphic sites are marked with green (common polymorphic site) or magenta (rare polymorphic site).
