# Supplementary material for: Allelic variations in the chpG effector gene within Clavibacter michiganensis populations determine pathogen host range
Source: PLoS Pathog. 2024 Jul 19;20(7):e1012380. doi: 10.1371/journal.ppat.1012380 (PMC11290698; doi:10.1371/journal.ppat.1012380)
Supplement: S7 Fig — Overnight cultures of Clavibacter michiganensis clones carrying pHN216 with the indicated inserts expressed under the pCMP1 promoter were diluted to OD600 = 0.5, lysed with protein sample buffer and separated on SDS-PAGE and gels were either stained with Coomassie brilliant blue (A, B, C, bottom panel) or transferred to nitrocellulose membrane and immunoblotted with anti-HA antibody (A, B, C, top panel). (PDF) [file ppat.1012380.s007.pdf]

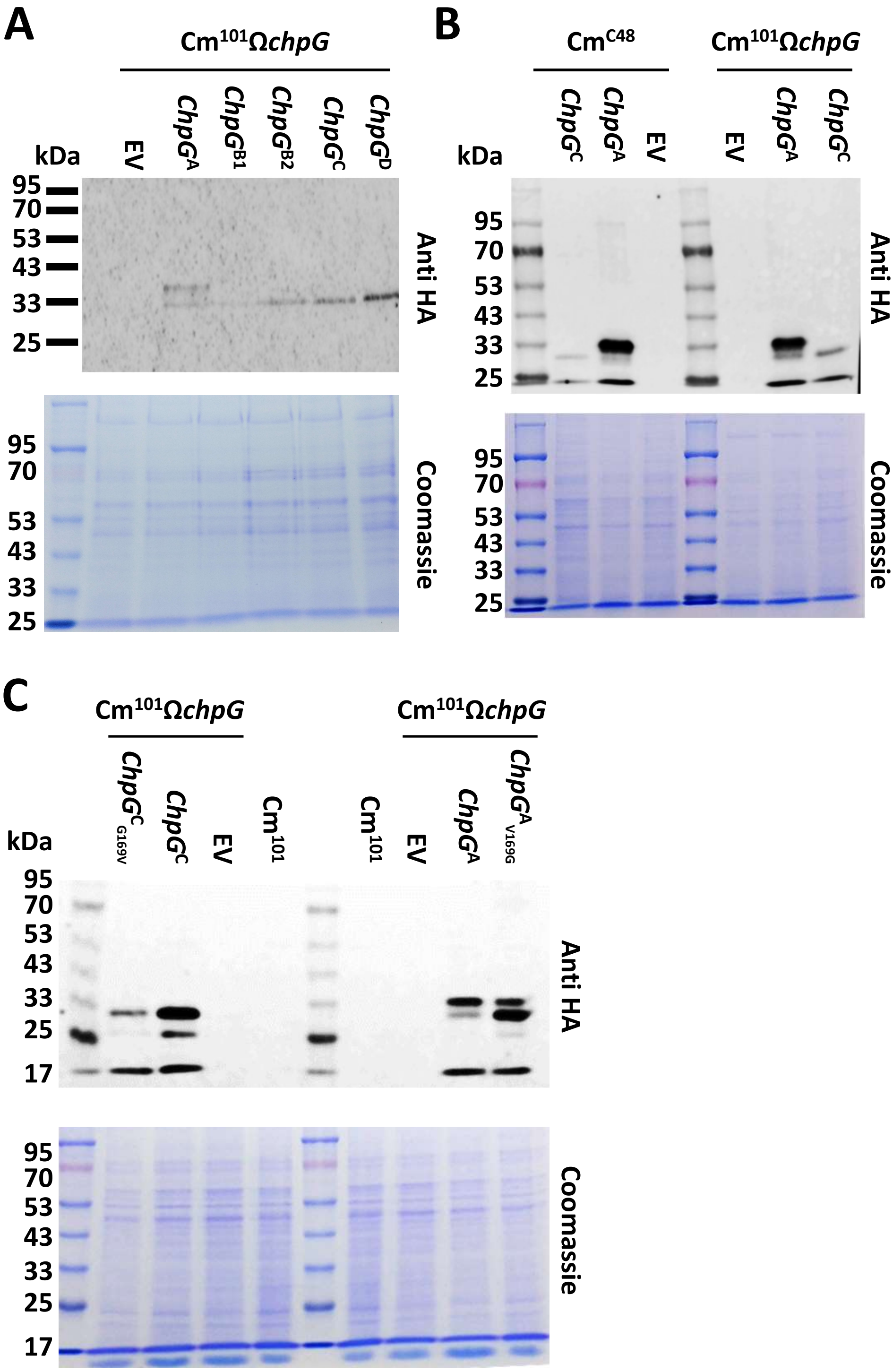

S7 Figure. Protein accumulation of ChpG variants in Cm^{101}ΩchpG and Cm^{C48}. Overnight cultures of Clavibacter michiganensis clones carrying pHN216 with the indicated inserts expressed under the pCMP1 promoter were diluted to OD_{600}=0.5, lysed with protein sample buffer and separated on SDS-PAGE and gels were either stained with Coomassie brilliant blue (**A**, **B**, **C**, **bottom panel**) or transferred to nitrocellulose membrane and immunoblotted with anti-HA antibody (**A**, **B**, **C**, **top panel**).
