# Supplementary material for: Allelic variations in the chpG effector gene within Clavibacter michiganensis populations determine pathogen host range
Source: PLoS Pathog. 2024 Jul 19;20(7):e1012380. doi: 10.1371/journal.ppat.1012380 (PMC11290698; doi:10.1371/journal.ppat.1012380)
Supplement: S8 Fig — Four-leaf stage "Moneymaker" tomato plants were inoculated with the indicated Clavibacter michiganensis (Cm) clones or water control (mock) by puncturing the stem area between the cotyledons with a wooden toothpick incubated in Cm solution (5 × 10^7^ CFU/ml). (A, B) Representative plants were photographed 21 days post inoculations (dpi). (C) Wilting symptoms were quantified at 21 dpi according to the following scale: 0 = no wilting, 1 = 1–25%, 2 = 25–50%, 3 = 50–100%. Graph depicts the symptom distribution in at least nine plants pooled from two independent experiments. (D) Stem bacterial populations 1 cm above the inoculation site at 21 dpi. Lower and upper quartiles are marked at the margins of the boxes. Central lines and “o” represent medians and data points of at least nine biological repeats collected from two independent experiments. (PDF) [file ppat.1012380.s008.pdf]

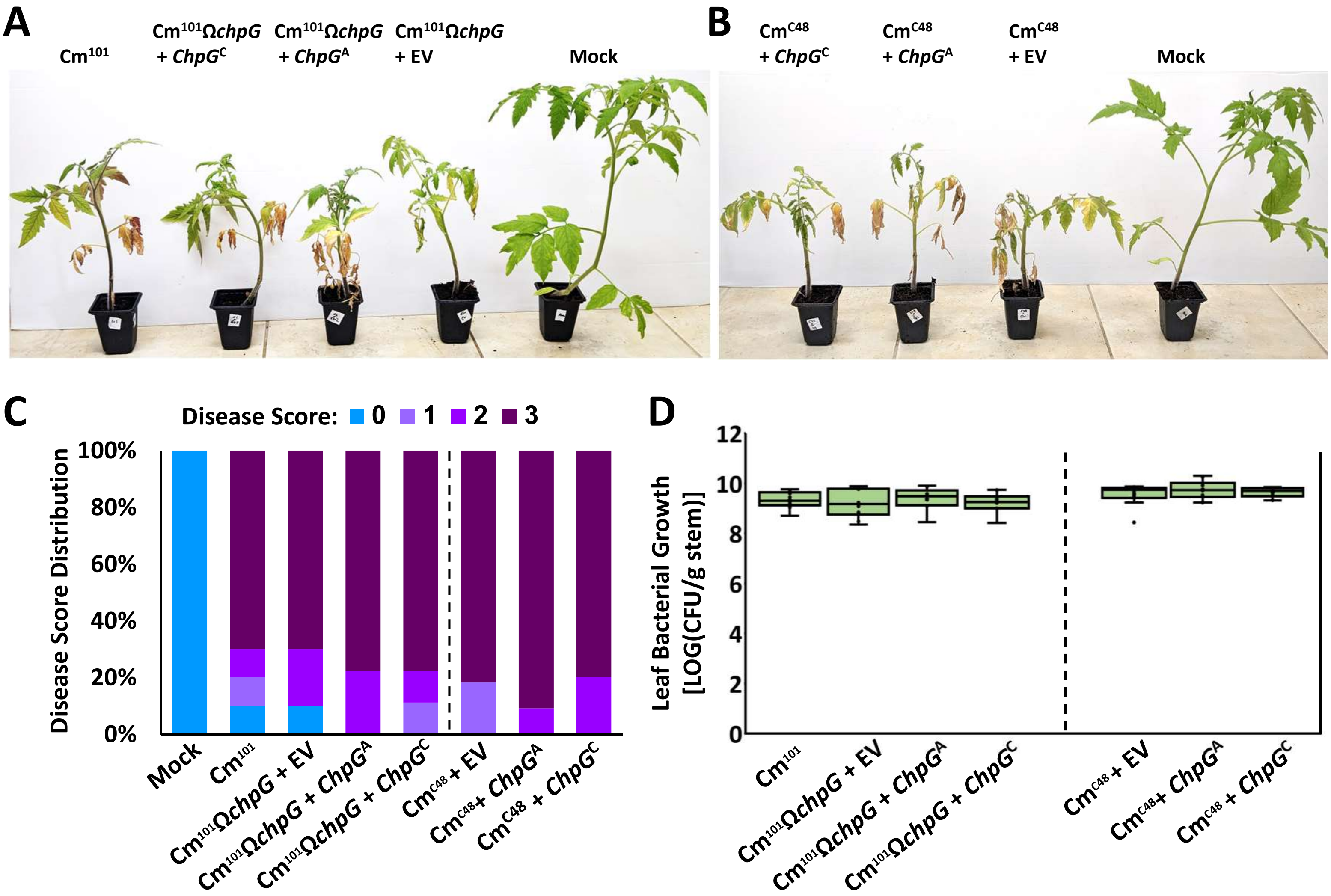

S8 Figure. Introduction of ChpG^{A} and ChpG^{C} into Cm^{C48} or Cm^{101}ΩchpG does not affect virulence on tomato. Four-leaf stage "Moneymaker" tomato plants were inoculated with the indicated *Clavibacter michiganensis* (Cm) clones or water control (mock) by puncturing the stem area between the cotyledons with a wooden toothpick incubated in Cm solution (5×10^7 CFU/ml). (**A**, **B**) Representative plants were photographed 21 days post inoculations (dpi). (**C**) Wilting symptoms were quantified at 21 dpi according to the following scale: 0 = no wilting, 1 = 1-25%, 2 = 25-50%, 3 = 50-100%. Graph depicts the symptom distribution in at least nine plants pooled from two independent experiments. (**D**) Stem bacterial populations 1 cm above the inoculation site at 21 dpi. Lower and upper quartiles are marked at the margins of the boxes. Central lines and “o” represent medians and data points of at least nine biological repeats collected from two independent experiments.
