# Supplementary material for: Allelic variations in the chpG effector gene within Clavibacter michiganensis populations determine pathogen host range
Source: PLoS Pathog. 2024 Jul 19;20(7):e1012380. doi: 10.1371/journal.ppat.1012380 (PMC11290698; doi:10.1371/journal.ppat.1012380)
Supplement: S9 Fig — (A) The 3D-structures of ChpGA (marked in aqua) and ChpGAV169G (marked in green) were predicted using Alpha-Fold2 through the AlphaFold Colab notebook platform. Structure alignment was produced using PyMOL. Enlarged rectangle represent structure shift in the 161–170 aa region. The 169 position is marked in magenta. (B) Root Mean Square Fluctuation (RMSF), representing the level fluctuation of individual residues from their average positions over a simulation trajectory of ChpGA (aqua) and ChpGAV169G (green) using the online WEBGRO Macromolecular Simulations server (https://simlab.uams.edu/ProteinWithLigand/protein_with_ligand.html). Simulation was repeated twice with similar results. (PDF) [file ppat.1012380.s009.pdf]

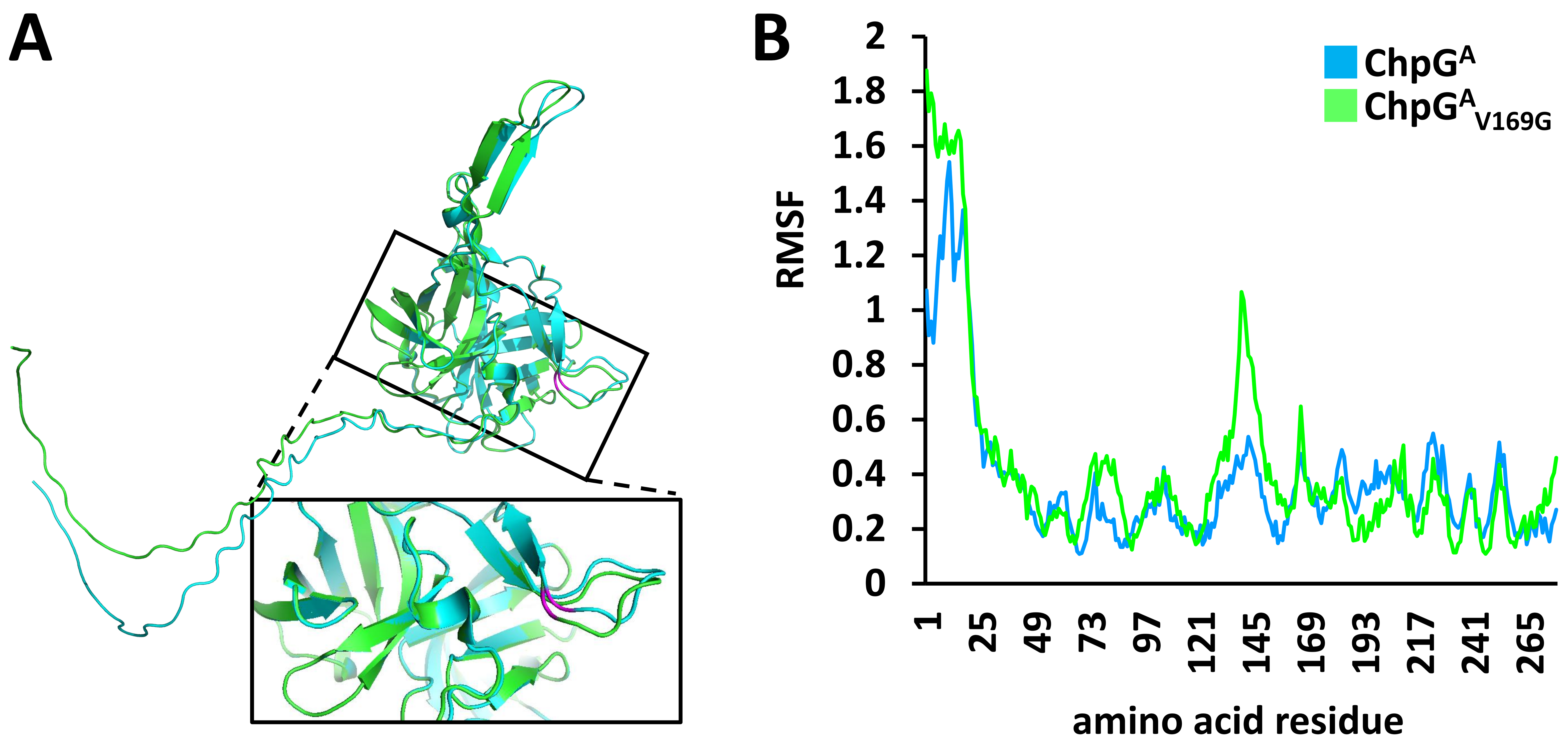

S9 Figure. Comparative analyses of the predicted 3D structures of ChpG^A and ChpG$^A_{V169G}$. (**A**) The 3D-structures of ChpG^A (marked in aqua) and ChpG$^A_{V169G}$ (marked in green) were predicted using Alpha-Fold2 through the AlphaFold Colab notebook platform. Structure alignment was produced using PyMOL. Enlarged rectangle represent structure shift in the 161-170 aa region. The 169 position is marked in magenta. (**B**) Root Mean Square Fluctuation (RMSF), representing the level fluctuation of individual residues from their average positions over a simulation trajectory of ChpG^A (aqua) and ChpG$^A_{V169G}$ (green) using the online WEBGRO Macromolecular Simulations server (https://simlab.uams.edu/ProteinWithLigand/protein_with_ligand.html). Simulation was repeated twice with similar results.
