# Supplementary material for: Allelic variations in the chpG effector gene within Clavibacter michiganensis populations determine pathogen host range
Source: PLoS Pathog. 2024 Jul 19;20(7):e1012380. doi: 10.1371/journal.ppat.1012380 (PMC11290698; doi:10.1371/journal.ppat.1012380)
Supplement: S10 Fig — (A) Five to six leaf-stage ’Black Queen’ eggplant leaves were infiltrated (108 CFU/ml) with Cm101ΔPAI expressing the indicated chpG variants or an empty vector control (EV). A representative photograph was taken 48 hours post-infiltration. (B) ’Black Queen’ eggplant (left panel) and Nicotiana benthamiana (right panel) leaves were infiltrated with Agrobacterium strains carrying vectors aimed at transient expression of the indicated mature chpG variants fused to the 33 aa secretion signal of NtPR1 at the N-terminus under the expression of the CaMV35S promoter. The 33 aa secretion signal of NtPR1 alone was used as an empty vector control (EV). Representative photographs were taken 72 hours post-infiltration. Photographs represent at least 15 repeats with similar results taken from at least two independent experiments. (PDF) [file ppat.1012380.s010.pdf]

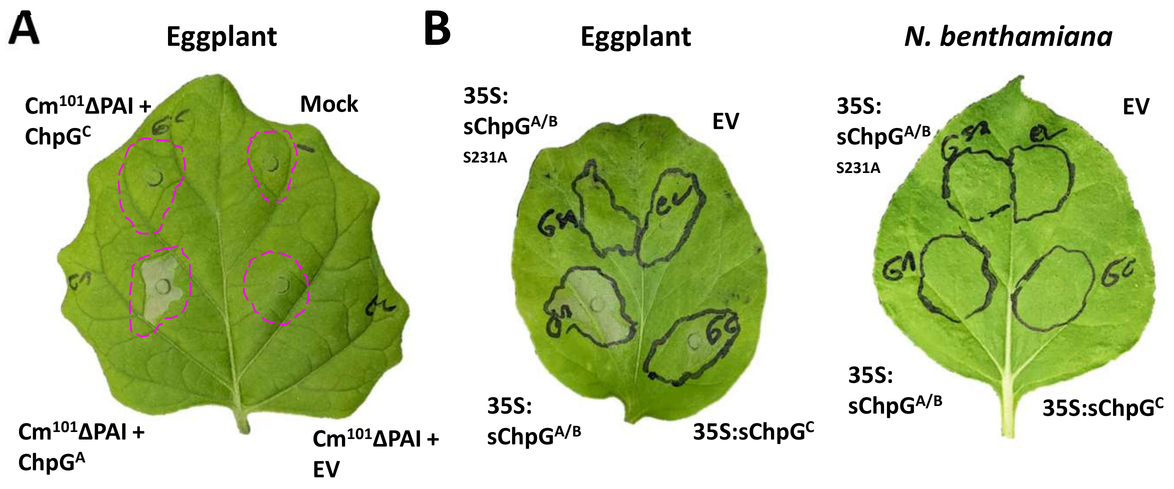

S10 Figure. Leaf infiltrations of Cm101ΔPAI and *Agrobacterium* carrying different *chpG* variants. (**A**) Five to six leaf-stage 'Black Queen' eggplant leaves were infiltrated (10^8 CFU/ml) with Cm^{101}ΔPAI expressing the indicated *chpG* variants or an empty vector control (EV). A representative photograph was taken 48 hours post-infiltration. (**B**) 'Black Queen' eggplant (**left panel**) and *Nicotiana benthamiana* (**right panel**) leaves were infiltrated with *Agrobacterium* strains carrying vectors aimed at transient expression of the indicated mature *chpG* variants fused to the 33 aa secretion signal of NtPR1 at the N-terminus under the expression of the *CaMV35S* promoter. The 33 aa secretion signal of NtPR1 alone was used as an empty vector control (EV). Representative photographs were taken 72 hours post-infiltration. Photographs represent at least 15 repeats with similar results taken from at least two independent experiments.
