# Supplementary material for: Allelic variations in the chpG effector gene within Clavibacter michiganensis populations determine pathogen host range
Source: PLoS Pathog. 2024 Jul 19;20(7):e1012380. doi: 10.1371/journal.ppat.1012380 (PMC11290698; doi:10.1371/journal.ppat.1012380)
Supplement: S1 Table — (DOCX) [file ppat.1012380.s011.docx]

**S1 Table. Cm clones used in this study**

| **Isolate name** | **Other name** | **Collection place** | **Collection region in Israel** | **Collection year** | **PFGE cluster** | **Plant/tissue of isolation** | **Source** | **Reference** |
| --- | --- | --- | --- | --- | --- | --- | --- | --- |
| Cm101 | CMM101 | Bielefeld University, Germany | NA | 1991 | NT^A^ | Lab strain | Rudolf Eichenlaub (Bielefeld University) | [1] |
| Cm^101^Ω*chpG* | CMM101chpGβ | Bielefeld University, Germany | NA | 2008 | NT | Lab strain | Rudolf Eichenlaub (Bielefeld University) | [2] |
| Cm^101^∆PAI | CMM30-18 | Bielefeld University, Germany | NA | 2008 | NT | Lab strain | Rudolf Eichenlaub (Bielefeld University) | [3] |
| C3 | 62 | NA^B^ | Gaza strip | 1994 | N | Tomato plant | Shulamit Manulis-Sasson (Volcani Institute) | [4] |
| C4 | 63 | NA | Jordan valley | 1996 | O | Tomato plant | Shulamit Manulis-Sasson (Volcani Institute) | [4] |
| C5 | 29 | NA | Gaza strip | 2001 | L | Tomato seeds | Shulamit Manulis-Sasson (Volcani Institute) | [4] |
| C6 | 30 | Ahitov | Hefer valley | 2001 | M | Tomato fruit | Shulamit Manulis-Sasson (Volcani Institute) | [4] |
| C8 | 18 | Zipori | Lower Galilee | 1997 | C | Tomato plant | Shulamit Manulis-Sasson (Volcani Institute) | [4] |
| C18 | 150 | Avni Eithan | Golan heights | 2005 | D | Tomato plant | Shulamit Manulis-Sasson (Volcani Institute) | [4] |
| C20 | 88 | Netherlands | NA | NA | NC | Tomato plant | Shulamit Manulis-Sasson (Volcani Institute) | [4] |
| C21 | 32 | Talmi Eliyahu | North-Western Negev | 2001 | B | Tomato plant | Shulamit Manulis-Sasson (Volcani Institute) | [4] |
| C23 | 64 | NA | Gaza strip | 2000 | B | Tomato plant | Shulamit Manulis-Sasson (Volcani Institute) | [4] |
| C25 | 72 | Ami'oz | North-Western Negev | 2002 | B | Tomato plant | Shulamit Manulis-Sasson (Volcani Institute) | [4] |
| C26 | 110 | Mivtahim | North-Western Negev | 2003 | B | Tomato plant | Shulamit Manulis-Sasson (Volcani Institute) | [4] |
| C29 | 17 | Prigan | North-Western Negev | 2000 | A | Tomato plant | Shulamit Manulis-Sasson (Volcani Institute) | [4] |
| C30 | 127 | USA: Michigan | NA | NA | H | Tomato plant | Shulamit Manulis-Sasson (Volcani Institute) | [4] |
| C31 | 128 | USA: Ohio | NA | NA | H | Tomato plant | Shulamit Manulis-Sasson (Volcani Institute) | [4] |
| C32 |  | Maor | North Coastal Plain | 2019 | NT | Tomato plant | Ludmila Vagozeb (Israeli Plant Protection and Inspection Services) |  |
| C33 |  | Sde Yitzhak | North Coastal Plain | 2020 | NT | Tomato plant | Ludmila Vagozeb (Israeli Plant Protection and Inspection Services) |  |
| C34 |  | Sde Yitzhak | North Coastal Plain | 2020 | NT | Tomato plant | Ludmila Vagozeb (Israeli Plant Protection and Inspection Services) |  |
| C37 |  | Nitzanei Sinai | Central Negev | 2009 | D | Cherry tomato plant | Shulamit Manulis-Sasson (Volcani Institute) |  |
| C38 |  | Shaked | North Samaria | 2010 | D | Cherry tomato plant | Shulamit Manulis-Sasson (Volcani Institute) |  |
| C39 |  | Shekef | Lakhish Regional Council | 2010 | D | Tomato plant | Shulamit Manulis-Sasson (Volcani Institute) |  |
| C40 |  | Shibolim | North-Western Negev | 2010 | L | Tomato plant | Shulamit Manulis-Sasson (Volcani Institute) |  |
| C41 |  | Shibolim | North-Western Negev | 2011 | L | Tomato plant | Shulamit Manulis-Sasson (Volcani Institute) |  |
| C42 | 551 | Bikaat Arad | Northern Negev | 2011 | L | Tomato plant | Shulamit Manulis-Sasson (Volcani Institute) | [5] |
| C43 | 577 | Yesha | North-Western Negev | 2011 | K | Tomato plant | Shulamit Manulis-Sasson (Volcani Institute) | [5] |
| C44 | 182 | Hof Hacarmel | North Coastal Plain | 2007 | Z | Tomato plant | Shulamit Manulis-Sasson (Volcani Institute) | [5] |
| C45 | 259 | Mivtahim | North-Western Negev | 2009 | Z | Tomato plant | Shulamit Manulis-Sasson (Volcani Institute) | [5] |
| C46 |  | Ami'oz | North-Western Negev | 2010 | Z | Tomato plant | Shulamit Manulis-Sasson (Volcani Institute) |  |
| C47 |  | Mlilot | North-Western Negev | 2010 | Z | Tomato plant | Shulamit Manulis-Sasson (Volcani Institute) |  |
| C48 |  | Netiv HaAsara | North-Western Negev | 2011 | Z | Tomato plant | Shulamit Manulis-Sasson (Volcani Institute) |  |
| C49 | 183 | Dekel | North-Western Negev | 2007 | E | NA | Shulamit Manulis-Sasson (Volcani Institute) | [5] |
| C50 |  | Prigan | North-Western Negev | 2009 | E | Tomato plant | Shulamit Manulis-Sasson (Volcani Institute) |  |
| C53 |  | Ein Yahav | Central Negev | 2009 | B | NA | Shulamit Manulis-Sasson (Volcani Institute) |  |
| C54 |  | Ami'oz | North-Western Negev | 2009 | B | Tomato plant | Shulamit Manulis-Sasson (Volcani Institute) |  |
| C55 |  | Yesha | North-Western Negev | 2010 | B | Tomato plant | Shulamit Manulis-Sasson (Volcani Institute) |  |
| C56 |  | Mivtahim | North-Western Negev | 2011 | B | Tomato plant | Shulamit Manulis-Sasson (Volcani Institute) |  |
| C58 |  | Nitzanei Sinai | Central Negev | 2009 | A | Cherry tomato plant | Shulamit Manulis-Sasson (Volcani Institute) |  |
| C59 |  | Mivtahim | North-Western Negev | 2011 | A | Tomato plant | Shulamit Manulis-Sasson (Volcani Institute) |  |
| C61 |  | HaBsor experimental station | North-Western Negev | 2009 | NC | NA | Shulamit Manulis-Sasson (Volcani Institute) |  |
| C68 |  | Shekef | Lakhish Regional Council | 2011 | NC | Cherry tomato plant | Shulamit Manulis-Sasson (Volcani Institute) |  |
| C70 |  | Ami'oz | North-Western Negev | 2023 | NT | Tomato plant | Teper lab (Volcani Institute) |  |

^A^ NT - PFGE analysis was not conducted

^B^ NA - Collection year/place is unknown

^C^ NC- The isolate does not cluster to any group according to PFGE analysis

**References**:

1. Meletzus D, Bermphol A, Dreier J, Eichenlaub R. Evidence for plasmid-encoded virulence factors in the phytopathogenic *bacterium Clavibacter michiganensis* subsp. *michiganensis* NCPPB382. J Bacteriol. 1993;175: 2131–2136. doi:10.1128/jb.175.7.2131-2136.1993

2. Stork I, Gartemann K-H, Burder A, Eichenlaub R. A family of serine proteases of *Clavibacter michiganensis* subsp. *michiganensis* : *chpC* plays a role in colonization of the host plant tomato. Mol Plant Pathol. 2008;9: 599–608. doi:10.1111/j.1364-3703.2008.00484.x

3. Gartemann K-H, Abt B, Bekel T, Burger A, Engemann J, Flügel M, et al. The Genome Sequence of the Tomato-Pathogenic Actinomycete *Clavibacter michiganensis* subsp. *michiganensis* NCPPB382 Reveals a Large Island Involved in Pathogenicity. J Bacteriol. 2008;190: 2138–2149. doi:10.1128/JB.01595-07

4. Kleitman F, Barash I, Burger A, Iraki N, Falah Y, Sessa G, et al. Characterization of a *Clavibacter michiganensis* subsp. *michiganensis* population in Israel. Eur J Plant Pathol. 2008;121: 463–475. doi:10.1007/s10658-007-9264-z

5. Shtienberg D, Frenkel O, Rekah Y, Dror O, Abu-Moch F, Manulis-Sasson S. The prevalence, aggressiveness and survival of *Clavibacter michiganensis* subsp. *michiganensis* strains associated with different genetic groups in Israel. Acta Hortic. 2018; 303–310. doi:10.17660/ActaHortic.2018.1207.42
