# Supplementary material for: Allelic variations in the chpG effector gene within Clavibacter michiganensis populations determine pathogen host range
Source: PLoS Pathog. 2024 Jul 19;20(7):e1012380. doi: 10.1371/journal.ppat.1012380 (PMC11290698; doi:10.1371/journal.ppat.1012380)
Supplement: S2 Table — (DOCX) [file ppat.1012380.s012.docx]

**S2 Table: Sequencing statistics of Cm isolates sequenced during this study**

| Assembly | Total length | Total contigs | contigs (>= 1000 bp) | Largest contig | GC (%) | N50 | L50 | Accession |
| --- | --- | --- | --- | --- | --- | --- | --- | --- |
| C3 | 3219634 | 27 | 25 | 390595 | 72.87 | 285706 | 5 | JASBQC000000000 |
| C4 | 3239786 | 33 | 33 | 385376 | 72.91 | 176568 | 6 | JASBQB000000000 |
| C5 | 3295300 | 51 | 43 | 389381 | 72.77 | 152107 | 7 | JASBQA000000000 |
| C6 | 3266969 | 54 | 51 | 387924 | 72.8 | 126591 | 8 | JASBPZ000000000 |
| C8 | 3415064 | 63 | 44 | 555674 | 72.54 | 255269 | 5 | JASBPY000000000 |
| C18 | 3346254 | 45 | 41 | 439743 | 72.6 | 258731 | 5 | JASBPX000000000 |
| C20 | 3058618 | 50 | 38 | 390541 | 72.52 | 255249 | 5 | JASBPV000000000 |
| C21 | 3387424 | 46 | 41 | 328549 | 72.52 | 258771 | 6 | JASBPT000000000 |
| C23 | 3422864 | 50 | 44 | 356390 | 72.52 | 150424 | 7 | JASBPR000000000 |
| C25 | 3417812 | 44 | 39 | 384951 | 72.52 | 172337 | 6 | JASBPS000000000 |
| C26 | 3422263 | 45 | 39 | 458049 | 72.51 | 244537 | 5 | JASBPQ000000000 |
| C29 | 3296227 | 52 | 48 | 303660 | 72.75 | 110281 | 10 | JASBPP000000000 |
| C30 | 3247911 | 28 | 28 | 456785 | 72.76 | 176868 | 6 | JASBPU000000000 |
| C31 | 3156240 | 34 | 29 | 461445 | 72.69 | 281461 | 5 | JASBPN000000000 |
| C32 | 3296940 | 57 | 48 | 303660 | 72.75 | 110281 | 10 | JASBPM000000000 |
| C33 | 3289106 | 66 | 56 | 387672 | 72.79 | 107118 | 10 | JASBPH000000000 |
| C34 | 3318102 | 51 | 37 | 422419 | 72.62 | 301029 | 5 | JASBPI000000000 |
| C37 | 3357653 | 56 | 49 | 359398 | 72.56 | 175004 | 7 | JASBPL000000000 |
| C38 | 3342280 | 50 | 40 | 388206 | 72.6 | 279224 | 5 | JASBPK000000000 |
| C39 | 3347921 | 50 | 45 | 392497 | 72.6 | 134904 | 7 | JASBPF000000000 |
| C40 | 3289939 | 48 | 41 | 406300 | 72.79 | 220744 | 5 | JASBOY000000000 |
| C41 | 3288693 | 63 | 56 | 387672 | 72.79 | 107118 | 10 | JASBPE000000000 |
| C42 | 3288693 | 63 | 56 | 387672 | 72.79 | 107118 | 10 | JASBPD000000000 |
| C43 | 3302361 | 42 | 36 | 389694 | 72.75 | 252162 | 5 | JASBPC000000000 |
| C44 | 3364495 | 67 | 48 | 422297 | 72.65 | 154368 | 6 | JASBPB000000000 |
| C45 | 3347426 | 50 | 41 | 397013 | 72.62 | 297543 | 5 | JASBPA000000000 |
| C46 | 3385853 | 51 | 36 | 360537 | 72.59 | 229199 | 6 | JASBOZ000000000 |
| C47 | 3272850 | 54 | 41 | 360499 | 72.66 | 199457 | 6 | JASBOS000000000 |
| C48 | 3385061 | 65 | 44 | 390999 | 72.59 | 259622 | 6 | JASBOU000000000 |
| C49 | 3080482 | 88 | 67 | 367749 | 72.49 | 114849 | 8 | JASBOV000000000 |
| C50 | 3342280 | 50 | 40 | 388206 | 72.6 | 279224 | 5 | JASBOR000000000 |
| C53 | 3317376 | 46 | 37 | 422419 | 72.62 | 301029 | 5 | JASBOX000000000 |
| C54 | 3364907 | 42 | 34 | 636165 | 72.56 | 180294 | 5 | JASBOT000000000 |
| C55 | 3418633 | 51 | 41 | 576639 | 72.51 | 171727 | 6 | JASBOQ000000000 |
| C56 | 3346867 | 54 | 44 | 389053 | 72.62 | 203484 | 6 | JASBOL000000000 |
| C58 | 3249338 | 48 | 42 | 354034 | 72.77 | 136390 | 7 | JASBOO000000000 |
| C59 | 3292590 | 32 | 32 | 437340 | 72.76 | 218968 | 5 | JASBON000000000 |
| C61 | 3233702 | 43 | 40 | 341864 | 72.92 | 116463 | 8 | JASBOJ000000000 |
| C68 | 3351655 | 47 | 37 | 400823 | 72.58 | 222040 | 6 | JASBOK000000000 |
| C70 | 3398082 | 102 | 46 | 408569 | 72.6 | 153469 | 6 | JASBOM000000000 |
