# Supplementary material for: Allelic variations in the chpG effector gene within Clavibacter michiganensis populations determine pathogen host range
Source: PLoS Pathog. 2024 Jul 19;20(7):e1012380. doi: 10.1371/journal.ppat.1012380 (PMC11290698; doi:10.1371/journal.ppat.1012380)
Supplement: S3 Table — (DOCX) [file ppat.1012380.s013.docx]

**S3 Table. Primers used during this study**

| Primer name | Sequence (5’ to 3’) | Destination vector |
| --- | --- | --- |
| *Cloning primers* (bold underline represents restriction sites used for cloning) | | |
| ORFChpGF | AAA**GGATCC**ACGCTACGGAGGAACTCATGC | pMA-RQ:Cmp |
| ORFChpGR | CCC**TCTAGA**GTTGGCGGGTGCCTTGTAG | pMA-RQ:Cmp, pBTEX sHA |
| pMBPChpGF | AA**GGATCC**AACGGACTCAGCAACCCGG | pMALp5x, pBTEX sHA |
| pMBPChpGR | CC**GAATTC**GTTGGCGGGTGCGAGCTTG | pMALp5x |
| MCSHAF | AAA**GGTACC**GGATCCGTCGACCCCGGGC | pBTEX |
| MCSHAR | CCC**CTCGAG**GAGCTCGAATTCTCAAGCG | pBTEX |
| ssNtPR1F | AA**GGTACC**ATGGGATTTGTTCTCTTTTCACAATTGCCTTCATTTCTTCTTGTCTCTACACTTCTC | pBTEX |
| ssNtPR1R | GG**GGATCC**AGAATTTTGGGCACGGCAAGAGTGGGATATTACTAGGAATAAGAGAAGTGTAGAGAC | pBTEX |
| *Site directed mutagenesis primers* (bold underline represent nucleotide designated for substitution) | | |
| ChpGT506G-S | ACCGATCG**G**CGGGCGGCGAGCGATCGC | |
| ChpGT506G-AS | GCCGCCCG**C**CGATCGGTGAGGGCGGTG | |
| ChpGG506T-S | ACCGATCG**T**CGGGCGGCGAGCGATCGC | |
| ChpGG506T-AS | GCCGCCCG**A**CGATCGGTGAGGGCGGTG | |
| Amplification/sequencing primers from Cm genomic DNA | | |
| flaChpGF | CTCAGACAACACGACGAGGA | |
| flaChpGR | CTCTCCCTCACCAGACTCCA | |
