# Supplementary material for: Allelic variations in the chpG effector gene within Clavibacter michiganensis populations determine pathogen host range
Source: PLoS Pathog. 2024 Jul 19;20(7):e1012380. doi: 10.1371/journal.ppat.1012380 (PMC11290698; doi:10.1371/journal.ppat.1012380)
Supplement: S4 Table — (DOCX) [file ppat.1012380.s014.docx]

**S4 Table: Plasmids used in this study**

| **Plasmid** | **Relevant characteristic** | **Reference** |
| --- | --- | --- |
| pHN216 | *E. coli-Clavibacter* shuttle vector, based on the replicon of pCM2, Neo^R^/Km^R^ | [1] |
| pMA-RQ:Cmp | Synthetic construct carrying a HindII/EcoRI cassette containing pCMP1 promoter followed by a multiple cloning site a triple HA tag. Synthesized by GeneArt service Theromo-Fisher) | This study |
| pHN216:*ChpG*^A^-3×HA | pHN216 carrying *ChpG*^A^ (amplified from Cm^101^ strain, locus tag CMM_0059) ORF fused to triple HA-tag under the control of the pCMP1 promoter, Neo^R^/Km^R^ | This study |
| pHN216:*ChpG*^B1^-3×HA | pHN216 carrying *ChpG*^B1^ (amplified from C5 strain, locus tag EFE39_00385) ORF fused to triple HA-tag under the control of the pCMP1 promoter, Neo^R^/Km^R^ | This study |
| pHN216:*ChpG*^B2^-3×HA | pHN216 carrying *ChpG*^B2^ (amplified from C29 strain, locus tag LHJ47_00410) ORF fused to triple HA-tag under the control of the pCMP1 promoter, Neo^R^/Km^R^ | This study |
| pHN216:*ChpG*^C^-3×HA | pHN216 carrying *ChpG*^C^ (amplified from Cm C48 strain, locus tag QKF70_15430) ORF fused to triple HA-tag under the control of the pCMP1 promoter, Neo^R^/Km^R^ | This study |
| pHN216:*ChpG*^D^-3×HA | pHN216 carrying *ChpG*^D^ (amplified from C6 strain, locus tag QKG63_14905) ORF fused to triple HA-tag under the control of the pCMP1 promoter, Neo^R^/Km^R^ | This study |
| pHN216:*ChpG*^A^_V169G_-3×HA | pHN216:*ChpG* carrying a T506->G substitution within the ORF of *ChpG*^A^ (CMM_0059), Neo^R^/Km^R^ | This study |
| pHN216:*ChpG*^C^_G169V_-3×HA | pHN216:*ChpG* carrying a G506->T substitution within the ORF of *ChpG*^C^*,* Neo^R^/Km^R^ | This study |
| pHN216:*ChpG*^A^_S231A_-HA | pHN216:*ChpG* carrying a T691->G substitution within the ORF of *ChpG*^A^*,* Neo^R^/Km^R^ | [2] |
| pMALp5x | *E. coli* expression vector containing secreted maltose binding protein (MBP) tag, Amp^R^ | NEB (Ipswich,MA, USA) |
| pMALp5x:*ChpG*^A/B^ | pMALp5x carrying the 112-831 bp fragment (ORF minus signal peptide coding region) of *ChpG*^A^ or *ChpG*^B^ introduced into the BamHI/EcoRI sites, Amp^R^ | This study |
| pMALp5x:*ChpG*^C^ | pMALp5x carrying the 112-831 bp fragment (ORF minus signal peptide coding region) of *ChpG*^C^ introduced into the BamHI/EcoRI sites, Amp^R^ | This study |
| pMALp5x:*ChpG*^A/B^_S231A_ | pMALp5x carrying the 112-831 bp fragment (ORF minus signal peptide coding region) of *ChpG*^A^_S231A_ introduced into the BamHI/EcoRI sites, Amp^R^ | This study |
| pBTEX | Binary vector used for Agrobacterium-mediated transient expression, Km^R^ | [3] |
| pBTEX sHA | pBTEX carrying a cassette containing the the 33 aa signal peptide of *Nicotiana tabacum* PR1 protein (1-99 bp of accession num' X06930), MCS containing BamHI-SalI-SmaI-XbaI sites, and a triple HA tag, Km^R^ | This study |
| pBTEX: s*ChpG*^A/B^-3×HA | pBTEX sHA carrying the 112-831 bp fragment (ORF minus signal peptide coding region) of *ChpG*^A^ or *ChpG*^B^ introduced into the BamHI/XbaI sites, Km^R^ | This study |
| pBTEX: s*ChpG*^A/B^_S231A_-3×HA | pBTEX sHA carrying the 112-831 bp fragment (ORF minus signal peptide coding region) of *ChpG*^A^_S231A_ introduced into the BamHI/XbaI sites, Km^R^ | This study |
| pBTEX: s*ChpG*^C^-3×HA | pBTEX sHA carrying the 112-831 bp fragment (ORF minus signal peptide coding region) of *ChpG*^C^ introduced into the BamHI/XbaI sites, Km^R^ | This study |

*Neo^R^, Km^R^ and Amp^R^ indicate chloramphenicol, neomycin, kanamycin and ampicillin resistance, respectively

**References**:

1. Laine MJ, Nakhei H, Dreier J, Lehtilä K, Meletzus D, Eichenlaub R, et al. Stable transformation of the gram-positive phytopathogenic bacterium *Clavibacter michiganensis* subsp. *sepedonicus* with several cloning vectors. Appl Environ Microbiol. 1996;62: 1500–1506. doi:10.1128/AEM.62.5.1500-1506.1996

2. Verma RK, Teper D. Immune recognition of the secreted serine protease ChpG restricts the host range of *Clavibacter michiganensis* from eggplant varieties. Mol Plant Pathol. 2022;23: 933–946. doi:10.1111/mpp.13215

3. Frederick RD, Thilmony RL, Sessa G, Martin GB. Recognition Specificity for the Bacterial Avirulence Protein AvrPto Is Determined by Thr-204 in the Activation Loop of the Tomato Pto Kinase. Mol Cell. 1998;2: 241–245. doi:10.1016/S1097-2765(00)80134-3
